# Genome wide association study of 5 agronomic traits in olive (*Olea europaea* L.)

**DOI:** 10.1038/s41598-019-55338-w

**Published:** 2019-12-10

**Authors:** Hilal Betul Kaya, Deniz Akdemir, Roberto Lozano, Oznur Cetin, Hulya Sozer Kaya, Mustafa Sahin, Jenny L. Smith, Bahattin Tanyolac, Jean-Luc Jannink

**Affiliations:** 1 Department of Bioengineering, Faculty of Engineering, Manisa Celal Bayar University, Manisa, Turkey; 2 Cornell Statistical Consulting Unit, Cornell University, Ithaca, NY USA; 3 School of Integrative Plant Science, Plant Breeding and Genetics Section, Cornell University, Ithaca, NY USA; 4 Olive Research Institute, Izmir, Turkey; 5 National Clonal Germplasm Repository, USDA-ARS, One Shields Avenue, Davis, CA USA; 6 Department of Bioengineering, Faculty of Engineering, Ege University, Bornova, Izmir, Turkey; 7 USDA ARS, Robert W. Holley Center for Agriculture & Health, Ithaca, NY USA

**Keywords:** Plant breeding, Plant genetics

## Abstract

Olive (*Olea europaea* L.) is one of the most economically and historically important fruit crops worldwide. Genetic progress for valuable agronomic traits has been slow in olive despite its importance and benefits. Advances in next generation sequencing technologies provide inexpensive and highly reproducible genotyping approaches such as Genotyping by Sequencing, enabling genome wide association study (GWAS). Here we present the first comprehensive GWAS study on olive using GBS. A total of 183 accessions (FULL panel) were genotyped using GBS, 94 from the Turkish Olive GenBank Resource (TOGR panel) and 89 from the USDA-ARS National Clonal Germplasm Repository (NCGR panel) in the USA. After filtering low quality and redundant markers, GWAS was conducted using 24,977 SNPs in FULL, TOGR and NCGR panels. In total, 52 significant associations were detected for leaf length, fruit weight, stone weight and fruit flesh to pit ratio using the MLM_K. Significant GWAS hits were mapped to their positions and 19 candidate genes were identified within a 10-kb distance of the most significant SNP. Our findings provide a framework for the development of markers and identification of candidate genes that could be used in olive breeding programs.

## Introduction

Olive is among the most important trees worldwide. Current world production of table olive and olive oil is over 19.2^1^ and 3^2^ million tons, respectively. Olive tree products are some of the main components of the Mediterranean diet that contribute to good health^3^. Olive fruits and leaves contain various functional compounds, such as hydroxytyrosol and oleuropein, beneficial for human health^4,5^. Olive trees are predominantly located in Mediterranean and Asian countries though there is increasing cultivation in Argentina, the United States, Chile and Australia^1^ due to high consumption of olive products. It is thought that olive domestication began in the region closest to the border between Turkey and Syria about 6000 years ago, before olive cultivars spread throughout the Mediterranean countries via different routes^6^. Turkey has seen continuous cultivation of olive trees since then. In Turkey, olive growing regions occupy a large area including western and southern coastal strips of the country. This has led to the rich variety of cultivars in Turkey. In the long-term conservation efforts of plant genetic resources, olive germplasm collections play an important role^7^. There are more than 100 olive germplasm collections at international, national and regional levels in mostly Mediterranean countries for conservation and breeding purposes^8^. These collections have been extensively used in molecular studies including identification, molecular characterization and also mapping studies^9–11^.

In olive breeding programs, clonal selection and cross-breeding have been conducted for developing novel cultivars^12^, however, these efforts are slowed down by olive’s juvenile period and complex genome^13^. As a result, very few mapping studies have been reported for dissecting agronomic traits in olive and limited numbers of markers have been identified as related to fruit traits^14,15^, flower traits, tree growth traits such as trunk diameter^14^ and olive oil quality traits^16^.

Genome-wide association studies (GWAS), which emerged as an alternative to classical linkage mapping^17^, utilizes historical recombination in a diverse population^18^. Compared with QTL mapping, GWAS mostly provides a higher mapping resolution^19^ and does not need to use an experimentally developed segregating population. GWAS has evolved as a powerful tool to dissect the genetic architecture of complex traits in large germplasm sets. It does, however, require a large number of markers for whole genome scans in crops with low linkage disequilibrium (LD) and high haplotype diversity^20^.

Next generation sequencing (NGS) technologies have allowed discovering and genotyping thousands of markers in large and diverse germplasm collections^21^. Single-nucleotide polymorphisms (SNPs) have become popular in QTL mapping and GWAS in plants^20,22^. They are co-dominant and bi-allelic markers that are distributed along the whole genome^23^. Genotyping by Sequencing (GBS) is a simple and inexpensive technique originally developed for high-resolution association studies in maize^24^, which involves reducing genome complexity^25^ by using restriction enzymes. GBS has been implemented in many crops such as maize^24^, barley^26^, wheat^26,27^, soybean^28^, rice^29^, oat^30^ and cassava^31,32^ for purposes of genetic characterization, GWAS, linkage analysis and genomic selection. The GBS technique, which does not require prior knowledge of the genome, is preferred for species that do not have reference sequence information^25,30^. Genetic mapping with SNPs generated by GBS has been extensively used in tree species including peach^33^, grapevine^34^, sweet cheery^35^, eucalyptus^36^, oil palm^37^, and apple^38^, and found to be effective to identify marker traits associations. In olive, GBS technology was used to assess the genetic diversity in Italian cultivars^39^ and to construct linkage maps in F1^40–42^ and F2^43^ populations. However, there are no reports of using GBS based SNP markers for association mapping study in diverse olive accessions.

Most genetic studies in olive genotypes have focused on characterization of this species and QTL mapping^44^. GWAS in olive has been used by our group in Turkish olive genotypes using AFLP, SSR and SNP markers^45^. Here, we report the development and application of GBS in a diverse set of olive germplasm from Turkey and the USA. Our objectives were to (1) identify SNPs within olive genotypes based on GBS analyses and (2) perform a comprehensive GWAS to identify significant marker trait associations. Successful application of GBS in olive would suggest that the method can be used in other tree species.

## Results

### Evaluation of phenotypic data

**Figure 1:**
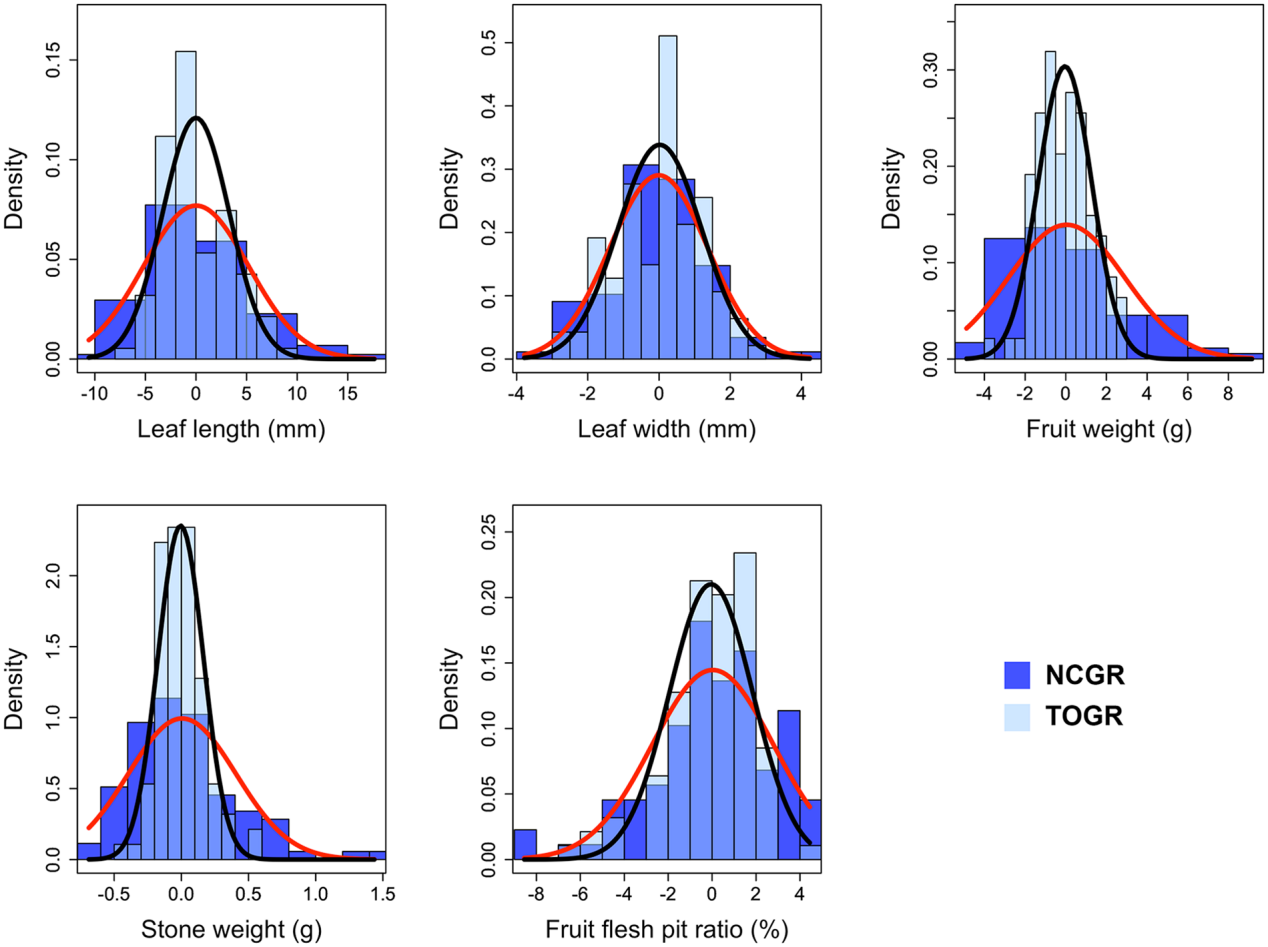
The distribution of BLUPs for phenotypic values used for the GWAS. Distribution of BLUPs of phenotypic values in TOGR (Turkish Olive GenBank Resource) and NCGR (National Clonal Germplasm Repository) panels. Black and red lines are normal distribution approximations for the TOGR and NCGR panels, respectively.

The descriptive statistics of leaf length (LL), leaf width (LW), fruit weight (FW), stone weight (SW) and fruit flesh pit ratio (FFPR) showed substantial variation was observed in all traits (Supplementary Table S1). Trait phenotypes ranged from 40.51 to 77.37 mm for LL, 7.30 to 26.10 mm for LW, 0.99 to 16.33 g for FW, 0.21 to 4.72 g for SW and 72.29 to 92.57 for FFPR. The statistical distribution of traits divided over geographical origin of accessions showed that year had a substantial effect on traits of accessions from NCGR, more so than TOGR (Supplementary Fig. S1). Relatively high *H*^2^ was calculated for FW and SW, 0.73 and 0.74, respectively (Supplementary Table S1). Heritability was moderate (0.52) for LW. The *H*^2^ estimates of LL (0.36) and FFPR (0.43) were low compared with other traits. The BLUPs of phenotypic values exhibited a near normal distribution for FULL, TOGR and NCGR panels (Fig. 1). Pearson’s correlation among the phenotypic traits showed that the highest degree of correlation was observed between FW and SW (r = 0.89). LL correlated positively with FW and SW (0.33 and 0.37, respectively). FW and SW also correlated positively with FFPR (0.6 and 0.28, respectively). There was no significant correlation between LW and other traits (Supplementary Fig. S2).

### Genotyping-by-sequencing and SNP detection

The fragment size distributions of GBS libraries from olive genomic DNA digested with *Eco*T22I and *Pst*I restriction enzymes are shown in Supplementary Fig. S3. The size distribution curve was smoother for *Eco*T22I (majority of fragments are <500 bp) than for *Pst*I and there was no highly repetitive DNA amplified (as would be evidenced by the presence of strong, discrete peaks or bands). The *Pst*I library, however, contained a large amount of repetitive DNA (discrete peaks/bands). *Eco*T22I was chosen for reducing genome complexity in olive. The sequencing produced a total of 469,721,669 raw reads, an average of 2.56 million reads per sample, and those reads produced 3,415,115 tags. A set of 61,892 unfiltered SNPs were obtained from the FULL panel. The percentage of missing data and minor allele frequencies for all accessions are shown in Supplementary Fig. S4. SNPs were filtered based on minor allele frequency (MAF > 0.05) and missing rate (<0.20). A final set of 24,977 SNPs were obtained and used for genetic diversity, population structure and GWAS.

### Genetic diversity and structure analysis

**Figure 2:**
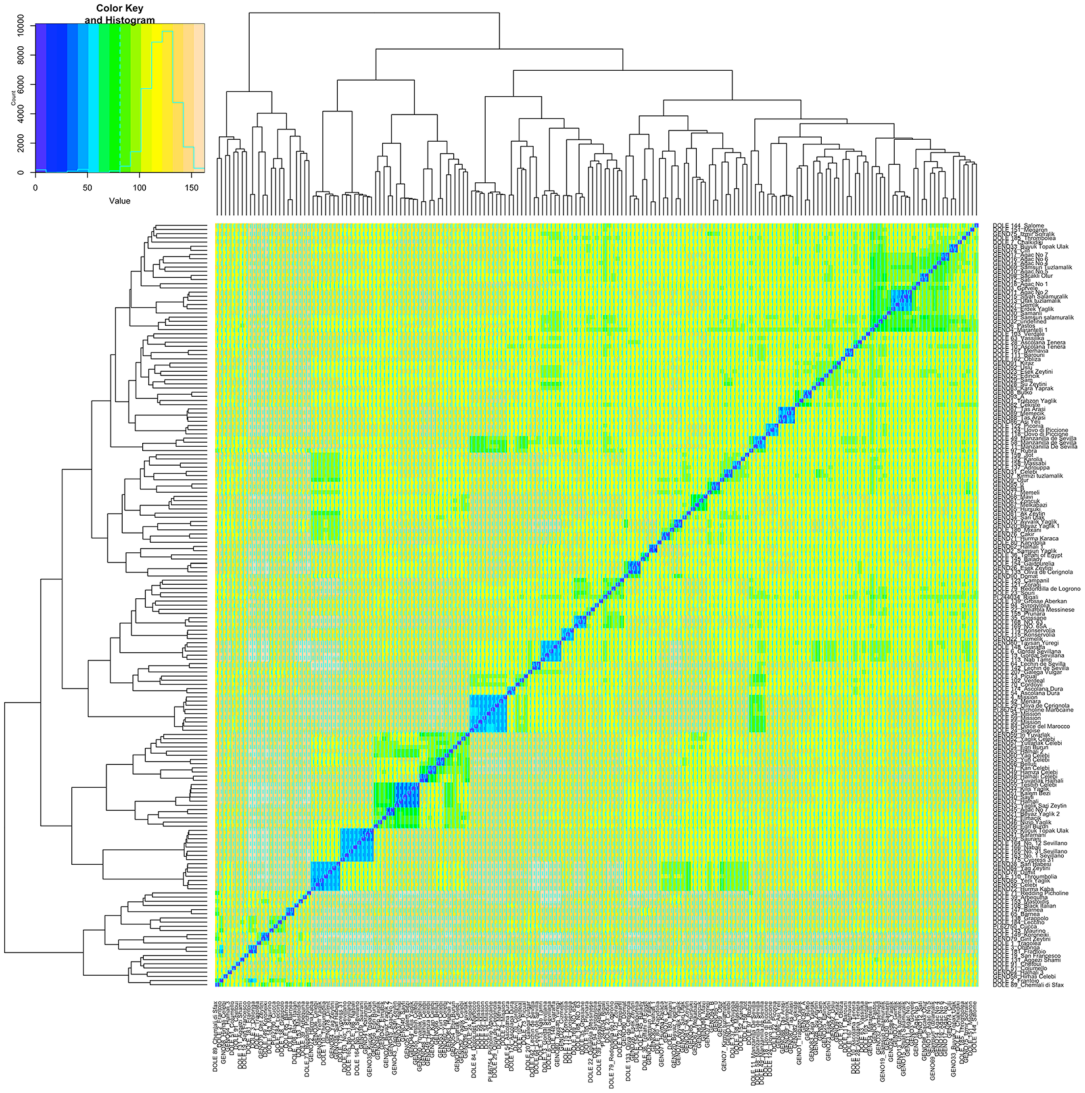
Heatmap based on genotypic pairwise Euclidean distances.

The genetic structure of the FULL panel was estimated using two complementary approaches. We estimated the marker-based kinship and found 67.2% of the kinship coefficients ranged from 0 to 0.2, indicating that most accessions have weak genetic relationship with the other accessions. This wide genetic diversity among olive accessions was also supported by their broader Euclidean genetic distance (28.74 to 162.28, mean: 120.61) (Supplementary Table S2). The maximum genetic dissimilarity between genotypes was 162.28 for Samsun Yaglik (GENO2) and Halhali 1 (GENO62). The minimum genetic dissimilarity was 28.74 for Gordal Sevillana (DOLE 13) and Koroneiki (DOLE 149). The genetic relationship for the FULL panel was visualized in the heatmap of the distance matrix (Fig. 2).

**Figure 3:**
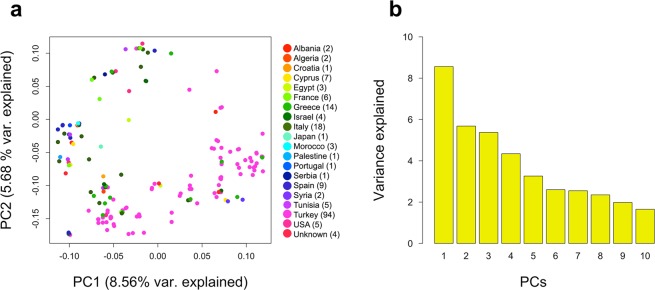
PCA of olive FULL panel a) Scatter plot of the first two principal components (PC1 and PC2). (**a**) The genetic variation explained by the first ten 10PCs (**b**).

PCA of the SNP dosage matrix was used to assess the clustering of genetic variation in olive accessions (Fig. 3a). PC1 explained 8.56% of the variation in the genotypic data, whereas PC2 and PC3 explained 5.68% and 5.37% of the variation, respectively (Fig. 3b). Although PCA analysis did not sort accessions based on their geographical locations, a subtle geographical pattern of distribution among Turkish genotypes may be deduced.

The optimum number of clusters (K) in the population was inferred to be six based on maximum likelihood and delta K (ΔK) values (Supplementary Fig. S5). When using a probability of membership threshold of 70%, 106 accessions were assigned into the six subgroups (Supplementary Fig. S6), while the remaining 77 accessions were classified into a mixed subgroup (Supplementary Table S3). Most accessions of Group 1 came from NCGR with only 2 from TOGR. Group 2 had the highest number of accessions among the groups with 23 and 22 accessions from TOGR and NCGR, respectively. The accessions of Group 3 were only from TOGR while the accessions of Group 6 included only accessions from NCGR. The accessions of Group 4 were primarily from TOGR with just six accessions from NCGR. Group 5 contained the fewest genotypes, with one from TOGR and four from NCGR. The Mixed group contained accessiong from both TOGR (36 accessions) and NCGR (41 accessions).

To investigate the extent of population differentiation between groups from STRUCTURE analysis, Fst values were calculated using the filtered markers (Supplementary Fig. S7). Genetic differentiation was higher between Group 5 and Group 6 (F_st_ = 0.417). The lowest degree of differentiation was found between Group 2 and Group 4 (F_st = _0.170). Fst values between the groups suggested that there was significant divergence across all groups.

### Linkage disequilibrium

A total of 20,799 (4.81%) pairs of markers showed a significant LD value (D’) at P < 0.01 while 2,678 pairs of markers showed a significant LD at *P* < 0.001. Based on *r*^2^ estimates, 28.4% and 14.7% of the marker pairs showed a significant LD value of *r*^2^ ≥ 0.05 and *r*^2^ ≥ 0.01, respectively. The *r*^2^ values for all significant loci ranged from 0.05 to 1. The mean *r*^2^ and *D’* for all pairs was 0.05 and 0.01, respectively. Supplementary Fig. S8 shows the distribution of the *r*^2^ values of all (left panel) and *r*^2^ ≥ 0.1 (right) for all marker pairs.

### Genome-wide association study

**Figure 4:**
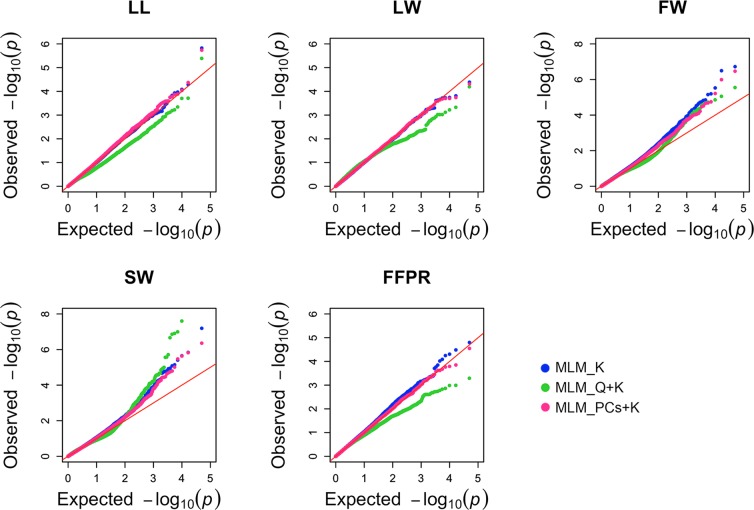
Comparison of QQ plots obtained with different GWAS models for five traits in the FULL panel.

Of the 3 three statistical models tested, the MLM_PCs + K model and the MLM_K model had similar power and showed a significant improvement in goodness of fit compared with the MLM_Q + K model. Increasing the number of PCs in the models did not decrease the type I error inflation (Supplementary Fig. S9). Hence, we kept only two PCs in the MLM_PCs + K model. Based on this information and on QQ plots of observed vs. expected P-values (Fig. 4), we chose the MLM_K model for association analysis and all subsequent results are based on it. Multiple testing burden was controlled using FDR correction^46^ at a 5% rate.

**Figure 5:**
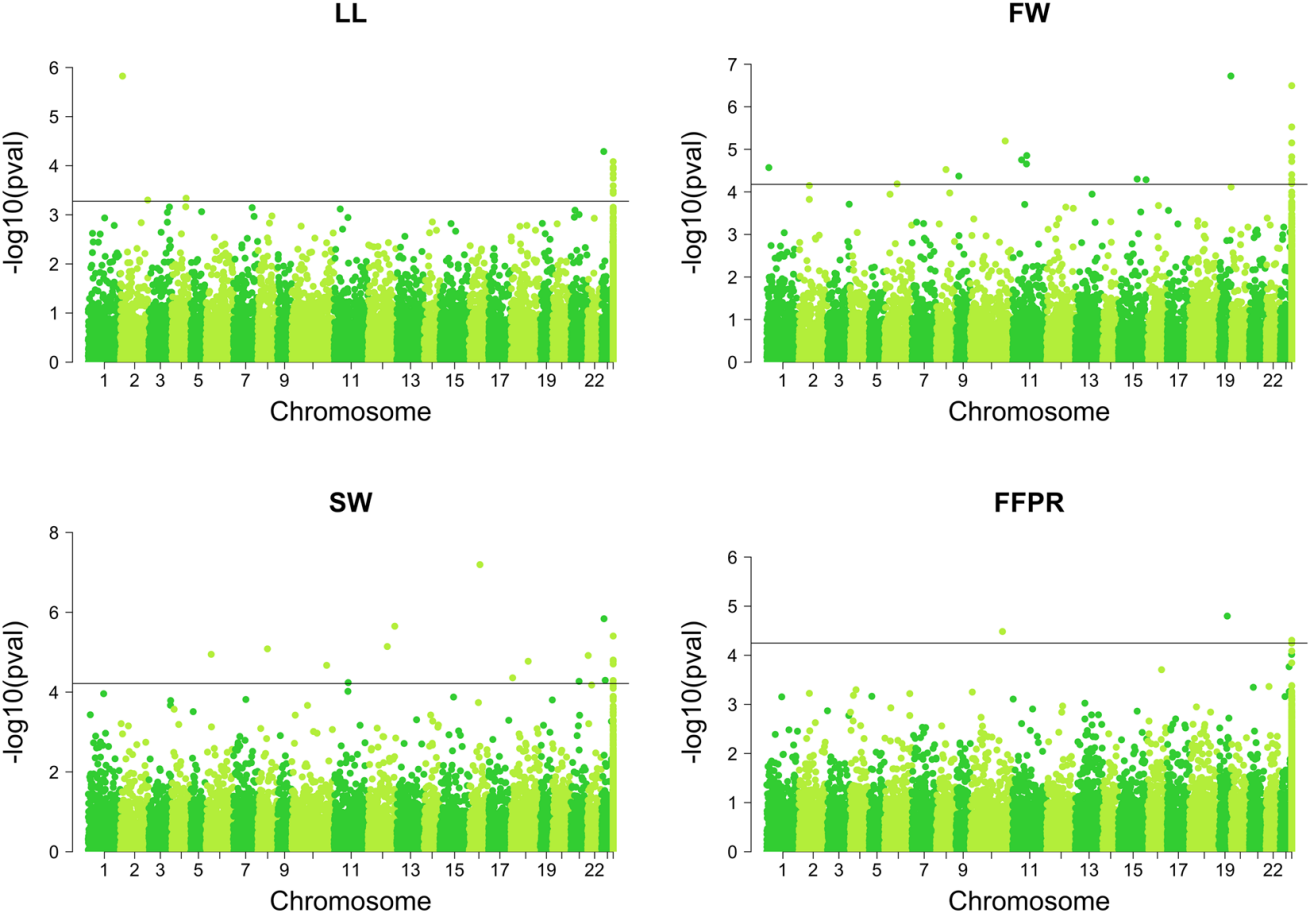
Manhattan plots summarizing genome-wide association results for LL, FW, SW and FFPR in the FULL panel. The FDR significance threshold is shown in black.

In total, 52 significant associations were detected in the FULL panel (Supplementary Table S4). Among these associations, 12, 19, 18 and 4 markers were associated with LL, FW, SW and FFPR respectively. No significant associations were detected for LW. The data listed in Supplementary Table S4 also showed that some of the markers were associated with more than one trait, e.g., S1_904125, S1_12591134, S1_1899635, S1_4122458 and S1_9030959 markers were associated with FW and SW. The most significant marker (S1_13767032) had a P-value of 9.11E-08 and was associated with SW (Fig. 5).

The twelve significant SNPs, which were identified for LL, explained 13.7% of phenotypic variance on average (8.5–20.4% for different loci). FF exhibited 19 significant SNPs that explained 5.27–27.8% of the phenotypic variance with an average 15.08%. A total of 18 significant SNPs was identified for SW, which explained 6.29–27.4% of the phenotypic variance with an average 13.18%. Variance explained by significant SNPs for FFPR ranged from 11.6 to 18.6% with an average of 14.7%.

Two recently published olive genomes were used to align sequence reads of significant SNPs. Out of the 53 significant associations in our study, 27 SNPs (51%) were mapped in the wild olive reference genome^41^ while 40 SNPs (75%) were mapped in the genome of *Olea europaea* L. subsp. *europaea* var. *europaea cv. ‘Farga’*^47^ (Supplementary Tables S5 and S6). Chromosomal positions of significant SNPs determined according to wild olive reference genome^41^ located SNPs far from each other. Four SNPs associated with LL were identified on chromosomes 2, 4 and 23. The 8 SNPs associated with FW were present on chromosomes 1, 6, 9, 11, 15 and 19 while the 10 SNPs associated with the SW were located on chromosomes 6, 12, 16, 18, 21, 22 and 23. Lastly, the 2 SNPs associated with the FFPR content were present on chromosomes 10, and 19. Among the significant SNPs, 5 were associated with both FW and SW. Three of them were mapped on chromosomes 8, 10, and 11. The most significant SNP marker (S1_13767032) explaining 19.8% of the phenotypic variance was located on chromosome 16 (Supplementary Table S5).

**Figure 6:**
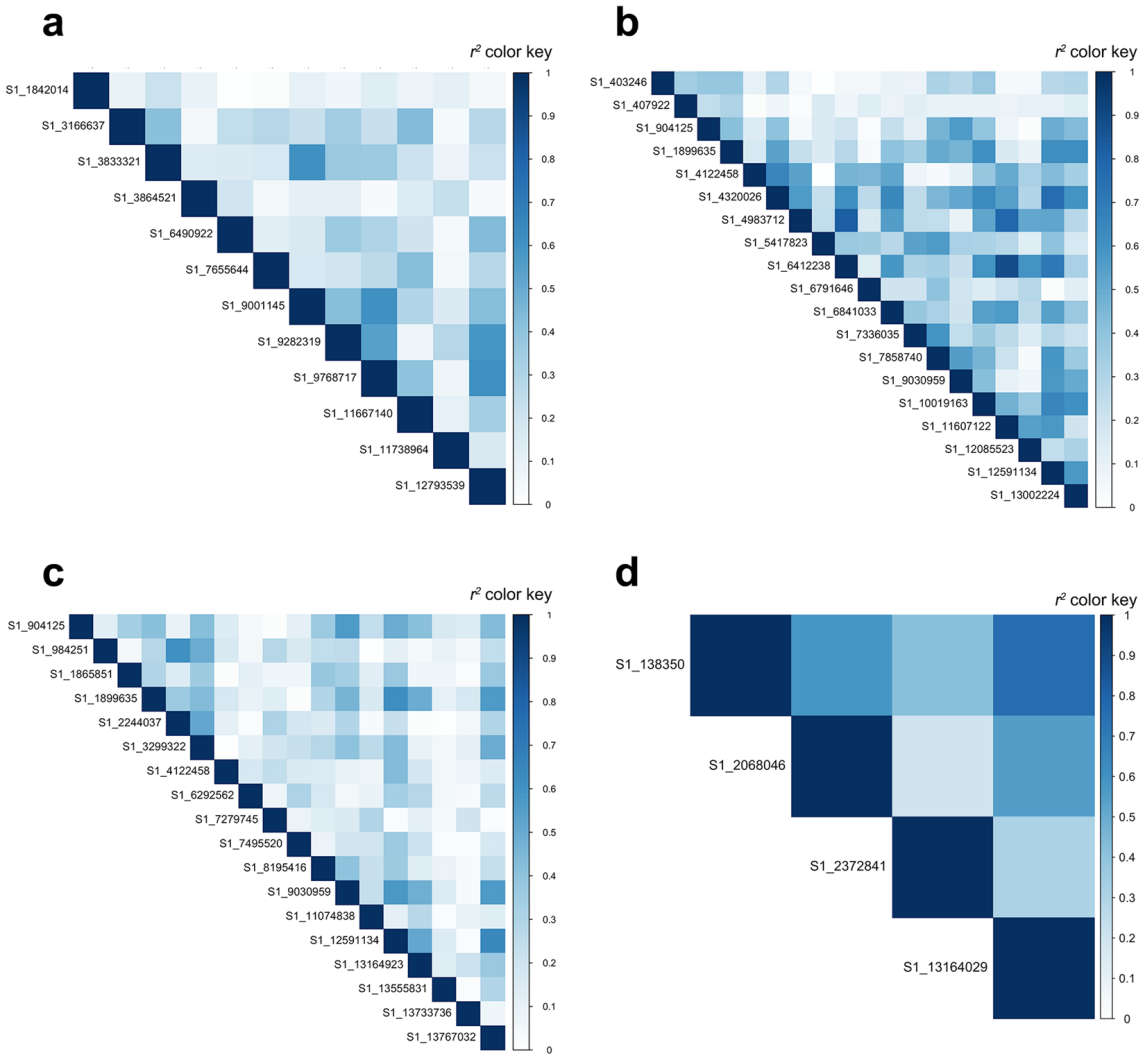
A heatmap of LD (*r*^2^) between significant SNPs, where darker blue colors represent stronger positive correlations between SNPs, (**a**) LL, (**b**) FW, (**c**) SW, (**d**) FFPR.

To assess the extent of association mapping, triangle plots for pairwise LD between significant markers were created for each trait (Fig. 6). The pattern of LD blocks shows that significant LD was not only detected between significant markers located on same chromosomes but also between significant markers on different chromosomes (chromosome information in Supplementary Table S5). The highest LD was obtained between two significant markers (S1_6412238 and S1_11607122, *r*^2^ = 0.88) associated with FW which were not aligned to the wild olive reference genome.

In the TOGR and NCGR panels, GWAS detected a total of 15 and 23 significant SNPs respectively (Supplementary Table S7 and S8, Supplementary Figs. S10 and S11). No significant SNP was detected for LW in either panel. The most significant SNP marker (S1_4640124) in TOGR panel had a P-value of 1.82e-05 and explained 25.5% of the phenotypic variation. Out of the 15 significant associations in TOGR panel, 7 SNPs (47%) were mapped in the wild olive reference genome (Supplementary Table S9)^41^ while 14 SNPs (93%) were mapped in genome of *Olea europaea* L. subsp. *europaea* var. *europaea cv. ‘Farga’* (Supplementary Table S10)^47^. In the NCGR panel, the SNP marker (S1_13473561) was the most significant SNP with a P-value of 7.15e-07 and explained 18% of the phenotypic variation. Twelve (52%) and 11 (48%) significant SNPs in NCGR panel were mapped in wild olive reference genome^41^ (Supplementary Table S11) and genome of *Olea europaea* L. subsp. *europaea* var. *europaea cv. ‘Farga’*^47^ (Supplementary Table S12) respectively.

### Candidate genes

Significant SNP tags were aligned against the wild olive reference genome. Regions within 10 kb were searched for candidate genes. A total of 19 unique genes were identified within these regions (Table 1), most of which are annotated to a protein that are responsible for developmental and physiological processes. For LL, two particular genes were found close to marker S1_1842014 on chromosome 2. We discovered 8 candidate genes for FW on chromosome 1, 9, 10, 11, 15 and 19. Two candidate genes for FW were present within a 2 kb window of S1_7336035 marker on chromosome 15. For SW, 8 genes were predicted on the chromosome 10, 12, 16, 18, 21 and 22 and three genes were found close to marker S1_6292562 on chromosome 22. On chromosome 10, 2 candidate genes were identified at upstream and downstream of S1_138350 marker that was significant for FFPR.

**Table 1:** List of the SNPs and nearest gene(s) for LL, FW, SW and FFPR.

Trait	Marker IDs	Nearest gene(s)	Location of gene(s)	Distance to SNP (kb)	Description
LL	S1_1842014	Oeu008156.1, Oeu008157.1	chr2:29784048..29784539, chr2:29793765..29794750	upstream 1.346, downstream 7.817	No apical meristem protein, E3 ubiquitin-protein ligase
FW	S1_7858740	Oeu033444.1	chr1:2176890..2179212	upstream 0.223	Act domain-containing protein
FW	S1_403246	Oeu048296.1	chr9:3228595..3228900	downstream 0.141	Mitochondrial ATP synthase g subunit (ATP-synt_G)
FW	S1_12085523	Oeu014982.1	chr11:9787762..9791084	upstream 6.349	Nucleolar protein 58 (NOP58)
FW	S1_13002224	Oeu017663.1	chr11:15125620..15131290	upstream 2.537	Calponin homology and kinesin motor domain-containing protein-related
FW	S1_7336035	Oeu060693.1, Oeu060694.1	chr15:20313657..20314145, chr15:20316141..20318953	upstream 0.381, downstream 1.669	Polynucleotidyl transferase, ribonuclease h-like superfamily protein
FW	S1_10019163	Oeu025341.1	chr19:13106491..13106931	upstream 8.408	Zinc-binding in reverse transcriptase (zf-RVT)
SW	S1_11074838	Oeu054419.1	chr12:20699067..20703923	interior	bZIP transcription factor (bZIP_1)
SW	S1_13767032	Oeu046142.1	chr16:11635780..11640029	downstream 0.497	Phd finger transcription factor
SW	S1_984251	Oeu059021.1	chr18:18573156..18573656	downstream 2.256	Late embryogenesis abundant protein (LEA_3)
SW	S1_13164923	Oeu041791.1	chr21:8827612..8830227	upstream 4.166	Beta catenin-related armadillo repeat-containing
SW	S1_6292562	Oeu057828.1, Oeu057830.1, Oeu057831.1	chr22:669428..671698, chr22:679506..679862, chr22:682550..684409	upstream 5.820, downstream 1.928, downstream 4.972	Sodium-bile acid cotransporter, fimbrin/plastin, fimbrin/plastin
FFPR	S1_138350	Oeu048482.1, Oeu048483.2	chr10:34975051..34975731, chr10:34976473..34978039	upstream 0.839, downstream 50.20	L-ascorbate peroxidase 3, two-component sensor histidine kinase
FW, SW	S1_904125	Oeu040505.2	chr10:37947127..37953467	upstream 1.959	HIV Tat-specific factor 1 (HTATSF1)

## Discussion

Understanding the genetics behind fruit, endocarp and leaf related traits is a key element for the improvement of olive accessions for breeding purposes. This study is the first comprehensive report of association analysis on olive using GBS markers. We used a diverse panel of 183 olive accessions from two different Genbank resources (TOGR and NCGR) to identify significant markers associated with LL, LW, FW, SW and FFPR. The large variation in traits observed among accessions as well as the significant correlations between some traits indicates the large phenotypic trait diversity among accessions. Three traits (LL, FW and SW) showed a larger than 3-fold difference between minimum and maximum values (Table S1). Previous studies in cultivated and wild olive genotypes indicated similar high correlations between FW and SW^14,48^ and large variation in fruit, leaf and endocarp related traits^49^. Arias-Calderon *et al*.^50^ observed significant phenotypic variability in traits such as fruit weight, stone weight and flesh/stone ratio among progenies, which agrees with our findings. Phenotypic measurements were carried out for two and four years in TOGR and NCGR accessions, respectively and considerable phenotypic variation was found for each year in all traits (Supplementary Fig. S1). Similar year variations for fruit and endocarp related traits have also been reported in other olive studies^14,48^. BLUPs were used in GWAS to reduce environmental deviation in association analysis as suggested by Piepho *et al*.^51^.

The estimates of *H*^2^ for SW, FW, LW, FFPR, and LL were high to low, ranging from 0.74 to 0.36. SW and FW were among the highly heritable (0.74 and 0.73 respectively) traits in this study (Table S1). High heritability estimates are indicative of high quality of the data obtained^52^. Moderate to high *H*^2^ estimates of LW, FW and SW obtained in this study imply that these traits are under strong genetic control. Heritability estimates are critical in plant breeding and genetics, but experimental approaches are difficult to implement especially in long-lived plants such as trees^53^. Only a limited number of studies that estimate broad sense and narrow sense heritability of tree, fruit, endocarp and oil related traits have been published in olive^50,54–59^ and, consistent with our results, relatively high heritability estimates for fruit and endocarp characteristics were obtained. Arias-Calderon *et al*.^50^ reported high narrow sense heritability (0.82) while Fanizza *et al*.^58^ reported a moderate heritability estimate (0.6) for FW. Zeinanloo *et al*.^59^ obtained higher *H*^2^ estimates (0.85) for FFPR than we did, and they also obtained *H*^2^ estimates for FW (0.42) and SW (0.31). Contrary to our study, Padula *et al*.^55^ reported higher *H*^2^ estimates for FFPR than FW. These findings imply that accessions had abundant genetic variation and were suitable for marker-trait association mapping. Previous studies indicated that core collections of different numbers of olive genotypes from Genbank collections showed abundant phenotypic and genetic variation^9–11^. To investigate suitability of the olive core collections for association mapping studies, different sampling approaches and different numbers of genotypes from the World Olive Germplasm Bank (WOGB) in Cordoba, Spain^9,10^ and the WOGB in Marrakech, Morocco^11^ were analyzed. The studies reported that both core collections contained mostly Western Mediterranean cultivars^9,10^ but core collections with cultivars that reflect the full geographic distribution of olive^11^ are suitable for association mapping.

We present the first application of GBS in diverse olive accessions from two different Genbank resources. The few GBS studies in olive to date have focused on genotyping F_1_ and F_2_ individuals^40–43^ and Italian cultivars^39^. SNP calling in highly heterozygous species such as olive is more difficult than inbred lines^34,38^. The GBS protocol we implemented enabled the discovery of thousands of SNPs. The *Eco*T22I restriction enzyme was used for the reduction of genome sequence complexity. Ipek *et al*.^40^ used *Ape*KI while Unver *et al*.^41^ selected a combination of *Pst*I-*Mse*I restriction enzymes to perform GBS in olive. D’Agostino *et al*.^39^ used *Eco*T22I restriction enzyme in GBS of Italian cultivars. A total of 24,977 SNPs were obtained after filtering which is higher than the number of SNPs detected in other GBS studies in olive^40,41^. The average number of sequence reads per sample we obtained (2.56 million) was similar to what was reported in other olive studies by Ipek *et al*.^40^ (2.1 million) and D’Agostino *et al*.^39^ (2.6 million).

Analysis of the population structure and genetic relatedness between accessions in a GWAS has critical importance for elimination of spurious marker-trait associations^18,20^. The PCA visualization did not show separation of accessions into subpopulations on the basis of geographic origin (East, Central, and West Mediterranean Basin, North America and Japan). Predictably, most Turkish genotypes were clustered together however, some fell into clusters comprised of European genotypes from NCGR. Diez *et al*.^9^. reported an indistinct geographical pattern of distribution among olive accessions from WOGB in Cordoba. Contrary our study, Belaj *et al*.^10^ and El Bakkali *et al*.^11^ showed that PCA analyses clustered olive accessions based on their geographic origin (western, central, and eastern Mediterranean).

The FULL panel in this study was divided into 6 groups by STRUCTURE analyses (Supplementary Fig. S6). Seventy-seven accessions (42%) were categorized as admixed with varying levels of membership in the 6 groups. Previous research has reported genetic admixture on olive^60–63^. Differentiation between groups due to genetic structure was measured with Fst values and an Fst value greater than 0.15 can be considered significant^64^. The pairwise Fst values between all groups were higher than 0.15, indicating high genetic differentiation in our FULL panel. We observed concordance between distance-based cluster analysis (Fig. 2) and model-based STRUCTURE analysis (Supplementary Fig. S6). Neither cluster nor STRUCTURE analysis distinguished the accessions based on their geographic origin. The lack of concordance between geographic and genetic distance may come from olive trees being transported among ancient civilizations around the Mediterranean basin^65^.

Other studies on population structure and genetic diversity of olive also reported high genetic variation among olive accessions, supporting our findings^7,60,66,67^. Precise evaluation of population structure and genetic diversity of germplasm collections is crucial for not only GWAS studies but also for efficient management of accessions in terms of conservation of genetic variability. Similar to other fruit trees, use of synonyms and homonyms are among the most common issues in cultivar designation of olive. Synonyms and homonyms in olive were widely reported using various marker techniques such as AFLP, SSR, SNP^68,69^. Koehmstedt *et al*.^69^ and Barranco *et al*.^68^ stated that ‘Oblonga’ and ‘Frantoio’ accessions were synonymous according to their findings obtained using limited number of SSRs. In our study, two separate approaches (model-based STRUCTURE and distance-based clustering) located these accessions close to each other. Frantoio (Dole 181 from Albany) and Oblonga (Dole 3 from France) accessions were closer to each other than any other pair of accessions. Also, the other Frantoio accession (Dole 2 from Albany) was located in same cluster with those accessions. Finally, some accessions that had the same accession name but different code numbers (DOLE 118 and DOLE 124, DOLE 10 and DOLE 28, DOLE 114 and DOLE 115, DOLE 6 and DOLE 13, DOLE 54 and DOLE 174, DOLE 11, DOLE 49 and DOLE 58) collected from different locations were located side by side in the heatmap (Fig. 2).

Linkage disequilibrium between markers is one of the critical factors in association mapping studies since it provides information related to mapping resolution and strength^70^. Different descriptors of the amount of LD, D’ and r^2^, were estimated for every pairwise combination of SNPs. In all, 28.4% of the marker pairs showed significant LD at r^2^ > 0.05. To date, very few studies attempted to estimate LD in olive using various numbers of accessions. El bakkali *et al*.^11^ obtained significant LD scores in 59.5% and 26.5% of the pairwise comparisons analyzing different number of accessions. LD measured in Turkish Genbank accessions^45^ was quite low in terms of r^2^. Low LD scores in a small data set of olive accessions also reported by Reale *et al*.^71^. The mating system of the species is one the most important factors that affects LD^72^. The creation of new recombination leads to low LD in out-crossing species^70^. The low LD we observed is similar to those other outcrossing tree species such as conifers^73^, almond^74^, eucalyptus^75^ and coffee^76^.

Fruit weight, stone weight and pulp stone ratio are agronomically important traits in olive tree similar to other fruit trees^77^. After proving that olive leaves are also a rich source of secondary metabolites^78^, leaf traits have also been included among important traits in olive. Limited information exists in olive on QTL linked to agronomic traits including flowering related traits^15^, fruit related traits^14^, trunk diameter and oil content^14,16^. To the best of our knowledge only three studies have been reported on association mapping in olive for fruit related traits^45,79^, oil content^80^ and plant vigor^45^. In two of these studies only 18^79^ and 22^80^ olive accessions were used while a study published by our group^45^ included 94 olive accessions. We tested three models on the FULL panel. The MLM_K model and MLM_PCs + K showed a *similar* expected distribution of P-values. We used the MLM_K model since it showed a significant improvement in goodness of fit. The MLM_K model also has a shorter computational time and it does not need any additional steps such as obtaining population structure^81,82^. Comparisons of different statistical models for GWAS were also conducted in other tree species such as apple^83^, pine^84^, and almond^74^. Previous studies in almond^74^ and grapevine^85^ reported that The MLM model with kinship matrix had a better fit by controlling population structure and relatedness.

We found 53 significant markers associated with four traits in the FULL panel, including 12 associations with LL, 19 associations with FW, 18 associations with SW and 4 associations with FFPR. Two significant markers (S1_6412238 and S1_11607122) associated with FW were in high LD (*r*^2^ = 0.88, Fig. 6). The most significant marker (S1_13767032, P-value = 9.11E-08), associated with SW, was in relatively high LD with marker S1_12591134 (*r*^2^ = 0.65). GWAS was also conducted for TOGR and NCGR panels separately. Fifteen and 23 significant markers were detected in TOGR and NCGR panels, respectively. None of these were common with each other or with significant markers found in FULL panel.

We identified significant SNP marker locations using two recently published olive reference genomes. Among the 53 significant SNPs, 27 SNPs (51%) and 40 SNPs (75%) were mapped in the wild olive reference genome^41^ and the genome of *Olea europaea* L. subsp. *europaea* var. *europaea cv. ‘Farga’*^47^, respectively. We mapped more significant SNPs in genome of *Olea europaea* L. subsp. *europaea* var. *europaea cv. ‘Farga'*^47^ which is likely due to its higher genome coverage (95%) compared to wild olive reference genome (42%). However, this reference genome^47^ does not have chromosome assignments so mapped significant SNPs could not be assigned to a particular chromosome using this reference genome (Supplementary Table S6). Chromosomal positions of 27 significant SNPs according to wild olive reference genome^41^ located them far from each other. Some significant markers (S1_12085523 and S1_13002224, S1_11607122 and S1_7336035) associated with FW were found on chromosome 11 and 15, respectively. Also, 6 significant markers associated with SW were found on chromosomes 12 (S1_11074838 and S1_13555831), 18 (S1_984251 and S1_2244037) and 23 (S1_7495520 and S1_8195416). Although these pairs of markers were located on same chromosome, they were distant from each other. Comparison of chromosomal locations between this study and previously published studies could not be done due to the use of different molecular marker techniques. It is also important to explore whether significant markers we found are located in the same regions as in the previously reported QTL studies. Limited information exists in olive identifying QTLs linked to the traits analyzed. In a QTL mapping study published by Sadok *et al*.^15^, 8 QTLs linked to fruit weight were identified on different 7 linkage groups using ISSR, SSR and AFLP markers. In another QTL mapping study^14^, one QTL was identified for fruit weight on linkage group 17, while 3 QTLs were identified linked to pulp/stone ratio on linkage groups 10 and 17 using DArT-SSR markers.

We found 19 candidate genes close to significant markers in the FULL panel within a 10-kb region window in either direction of a significant SNP. The most significant SNP marker, S1_13767032, was located on 0.497 kb upstream of the Oeu046142.1 gene. This gene is annotated as a PHD finger transcription factor and the family to which this gene belongs, plays a key role in regulating plant growth and development^86^. The analysis of transcriptomes provides genomic resources for functional annotation to discover genes for olive breeding^87^. To date, several transcriptome studies have been performed for olive using different organs at different developmental stages^41,47,87–89^. There have been attempts to identify candidate genes associated with important traits such as plant architecture^90^ and juvenility^91^ in olive, but no putative candidate genes underlying QTL have been reported. More studies are still required to facilitate validation of these results in different olive populations.

## Materials and Methods

### Plant materials

**Figure 7:**
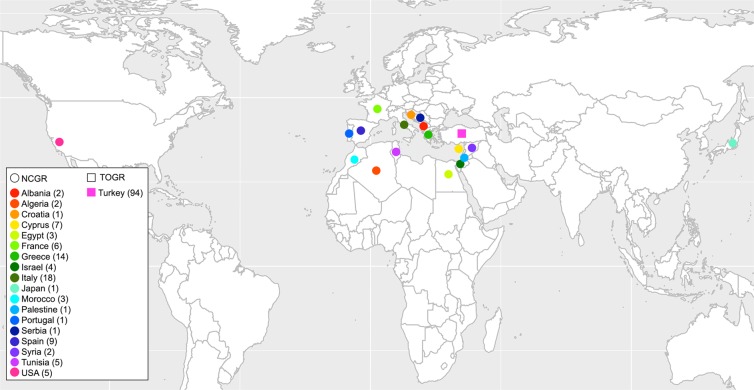
Geographical distribution of accessions. Origin of accessions is represented by a dot on the world map. Accessions without origin information (4 accessions from NCGR) are not shown in here.

We used 94 accessions from Turkish Olive GenBank Resources (TOGR) panel in Izmir, Turkey and 89 accessions from the USDA, ARS, National Clonal Germplasm Repository (NCGR) panel in Davis, CA, USA. Detailed information of these accessions (FULL panel) is provided in Fig. 7 and Supplementary Table S13. The map in Fig. 7 was generated using the ‘ggmap’ package^92^ in R version 3.4.2^93^. Fresh leaf tissue was harvested from the youngest leaves of each tree in the leaf shooting stage. Leaf tissue samples were stored at −80 °C until DNA was extracted.

### Phenotypic data

Phenotypic data for the following five traits was evaluated: leaf length (LL), leaf width (LW), fruit weight (FW), stone weight (SW) and fruit flesh to pit ratio (FFPR). For 94 accessions from TOGR, phenotypic data measurements (FW and SW) were carried out during 2011 and 2013 and this data was obtained from a previously published study by Kaya *et al*.^45^. LL, LW and FFPR measurements were carried out according to the methodology proposed by the International Olive Oil Council (IOOC). Phenotypic data for 89 accessions from NCGR in Davis was downloaded from USDA-GRIN (Germplasm Resources Information Network system) website for four seasons from 2005 to 2008 (Available at www.ars-grin.gov/npgs and accessed May 2017). To show the statistical distribution of traits divided over geographical origin of accessions and year, box plots were generated using R^93^. Phenotypic data from different years was averaged and used for descriptive statistical analysis using the pastecs package in R.^93^.

To eliminate the effect of environment variation, the best linear unbiased prediction (BLUP) values of lines were calculated for each trait using mixed linear model (1):

1 $${\bf{Y}}={\rm{X}}{\bf{b}}+{\rm{W}}{\bf{p}}+{\rm{Z}}{\bf{a}}+{\rm{e}}$$

where X, W and Z are incidence matrices, **b** is the vector of fixed effect for country and **p** and **a** are vectors of random effects for genotype and year, respectively. The random effects and residual errors are assumed to be normally distributed independent of each other and each of them have covariance structure proportional to an identity matrix. We fitted the model using the lmer function from the lme4 R package^94^. The predictions for the random effects for GIDs (BLUPs) from this model were used as phenotypic data for the GWAS.

Variance components were extracted from the lmer output and broad-sense heritability *(H*^2^), based on clone means, was estimated according to Hallauer *et al*.^95^. The *H*^2^ for each trait was estimated by the Eq. (2) using variance components from lmer.

2 $${H}^{2}={\rm{VG}}/({\rm{VG}}+{\rm{VE}})$$

where VG and VE represent estimates of genetic and environmental variance, respectively. In addition, heritability value estimates were calculated for TOGR and NCGR panels, separately. Pearson correlations between traits were estimated from BLUPs using the “cor” function in R and distributions of BLUPs in the two locations were plotted in R.

### DNA extraction and genotyping-by-sequencing

Genomic DNA was extracted by the CTAB method of Doyle^96^ with some minor modifications. The Chloroform: Isoamyl Alcohol (24:1) extraction step was applied twice to improve removal of phenolic compounds. DNA was quantified with Qubit dsDNA BR Assay Kit (Invitrogen) on a Qubit 2.0 Fluorometer (Invitrogen) according to the manufacturer’s instructions. DNA samples were diluted to 20 ng/μl and subsequently used for GBS library preparation.

The GBS libraries were constructed in 96-plex where each plate included a single random blank well as control. For choosing appropriate restriction enzymes for olive genotypes, two different restriction enzymes, *Eco*T22I (ATGCAT) and *Pst*I (CTGCAG) (both 6-base cutters), were tested to make GBS libraries and *Eco*T22I was selected. PCR amplification was performed to generate the GBS libraries and DNA was sequenced on an Illumina HiSeq. 2000 (Illumina Inc., USA). GBS was carried out at the Institute of Genomic Diversity (Cornell University, Ithaca, NY, USA) as described by Elshire *et al*.^24^.

### SNP calling, filtering and imputation

SNPs were identified using the TASSEL UNEAK (non-reference) GBS pipeline^97^ in the TASSEL 3 bioinformatics analysis package. SNPs were filtered to remove markers with more than 80% missing data and genotypes with more than 80% missing SNP calls in using R version 3.4.2^93^. After filtering, the SNP dataset was converted to numeric coding (1, 0, −1) from nucleotide coding for statistical analysis in R version 3.4.2^93^. The A.mat function from rrBLUP package in R was used to remove markers with minor allele frequency (MAF < 0.05) and impute the missing marker data based on expectation maximization (EM) algorithm.

### Genetic diversity and population structure analysis

To assess genetic structure, we applied both model-based and distance-based approaches. The Admixture-based clustering model we applied was STRUCTURE v.2.3.4^98^. Each simulation included 10,000 burn-in and 50,000 iterations. Ten independent runs were performed for each K value ranging from 1 to 10 with an admixture model and correlated allele frequencies. The optimal K was chosen based on Evanno’s methods^99^ using the STRUCTURE HARVESTER software^100^. To visualize the population structure, a bar plot was obtained with sort by Q option based on the optimum K value. Genotypes with membership probabilities higher than 0.7 were assigned to one of the subpopulations. Otherwise, they were considered to be admixed. The membership coefficient matrix (Q matrix) that shows the percentages of admixture of each accession given by the STRUCTURE software was used as cofactors in the association analyses. Calculation of pairwise genetic differentiation (Fst) between the groups from STRUCTURE was performed using VCFtools^101^. Principal component analysis (PCA) was also carried out to study the structure of the genotypes using the function prcomp in R^93^. First two principal components (PCs) were plotted using the ggplot2 R package^102^. To apply the distance-based approach, a Euclidean marker distance matrix was obtained using the dist function and a graphical representation of distance matrix was created using the heatmap function in R.

### Estimation of linkage disequilibrium

The LD between marker pairs was calculated based on D’ and *r*^2^ using TASSEL. Permutation testing was applied to examine the significance level of LD between loci. Pairwise LD analysis between significant markers for the best GWAS model based on marker score correlations (*r*^2^) was performed separately on the FULL panel and was visualized as a heatmap using heatmap function in R.

### Association analyses

Genome wide association analyses were performed using the SNP dataset consisting of 24,977 SNPs in FULL, TOGR and NCGR panels. For association analysis, three different models were tested for controlling population structure with the R package rrBLUP^103^. Mixed Linear Model (MLM) that accounts only for relative kinship (MLM_K model), MLM that accounts for both relative kinship and model-based population structure (MLM_Q + K) and MLM with first two PCs and K-matrix as correction for population structure (MLM_PCs + K) were compared. Q is the matrix of sub-population membership probabilities obtained from STRUCTURE^98^, K is the kinship matrix calculated using the A.mat function in the rrBLUP R package^103^ and the number of PCs was selected based on the scree plot of the variance explained by the first 10 PCs. We also examined the effect of including different numbers of PCs (first two PCs, first three PCs, first four PCs, first five PCs, first 10 PCs, first 15 PCs) as covariates in MLM_PCs + K model.

Correction for multiple testing was carried out using the false discovery rate (FDR) values according to the procedure by Benjamini and Hochberg^46^. Markers with FDR < 0.05 were considered significant. The proportion of phenotypic variance explained by each significant marker was estimated via R^2^ by fitting a regression between phenotypes and marker profiles using R^93^. The quantile–quantile (QQ) plots were used for selecting the best GWAS model. The QQ plots were produced using the R qqman package^104^ and Manhattan plots were visualized using R^93^.

### Mapping the significant hit to the olive reference genome

The significant GWAS hits were mapped to their positions in the two recently published olive genomes. The first genome, *Olea europaea* var. *sylvestris*,^41^ includes ~1.1 Gb of sequence and is available at https://phytozome.jgi.doe.gov. Only ~573 Mb of this assembly is mapped to its chromosomal position (n = 23). The second olive genome, *Olea europaea* L. subsp. *europaea* var. *europaea cv. ‘Farga’*^47^ has a total length of 1.31 Gb which represents 95% of the genome’s estimated size (1.38 Gb). This genome has no chromosome assignments and is divided in more than 50k scaffolds with an N50 of 443Kb.

Briefly, the significant GWAS hits were linked to their 64mer sequence tag using the TOPM file produced by the UNEAK GBS calling pipeline^97^. A multi-fasta was created using the marker identifier and the 64mer sequence. We then used Blast +^105^ to map each GWAS hit with its most probable location in both genomes. The best blast hit was chosen based on the percentage of alignment and E-value.

### Identification of candidate genes

To find candidate genes associated with significant SNPs, the Jbrowse feature of Phytozome v.12.1 (http://phytozome.jgi.doe.gov/pz/portal.html)^106^ was used to browse the wild olive reference genome^41^. Candidate genes were searched within 10 kb upstream and downstream of each significant SNP region in the genome browser.

## Supplementary information

Supplementary Information

Supplementary Table S2

Supplementary Table S3

Supplementary Table S13

## Data Availability

Raw FASTQ data have been submitted to the NCBI Short Read Achieve with accession number SRP113625.
