# Supplementary material for: Genome wide association study of 5 agronomic traits in olive (Olea europaea L.)
Source: Sci Rep. 2019 Dec 10;9:18764. doi: 10.1038/s41598-019-55338-w (PMC6904458; doi:10.1038/s41598-019-55338-w)
Supplement: Supplementary file 1 — Supplementary Information [file 41598_2019_55338_MOESM1_ESM.pdf]

Supplementary Information

Genome wide association study of 5 agronomic traits in olive (*Olea europaea* L.)

Hilal Betul Kaya[1,3*], Deniz Akdemir[2], Roberto Lozano[3], Oznur Cetin[4], Hulya Sozer Kaya[4], Mustafa Sahin[4], Jenny L. Smith[5], Bahattin Tanyolac[6], Jean-Luc Jannink[3,7]

[1] Department of Bioengineering, Faculty of Engineering, Manisa Celal Bayar University, Manisa, Turkey

[2] Cornell Statistical Consulting Unit, Cornell University, Ithaca, NY, USA

[3] School of Integrative Plant Science, Plant breeding and Genetics Section, Cornell University, Ithaca, NY, USA

[4] Olive Research Institute, Izmir, Turkey

[5] National Clonal Germplasm Repository, USDA-ARS, One Shields Avenue, Davis, CA, USA

[6] Department of Bioengineering, Faculty of Engineering, Ege University, Bornova, Izmir, Turkey

[7] United States Department of Agriculture, Agricultural Research Service (USDA-ARS) Ithaca, NY, USA

***Correspondence:**

Hilal Betul Kaya, e-mail: hilalbetul.kaya@cbu.edu.tr

Table of contents

Supplementary Fig. S1-S11

Supplementary Fig. S1. The box plots show the distribution of traits divided over geographical origin of accessions and year. TOGR (Turkish Olive GenBank Resource), NCGR (National Clonal Germplasm Repository).

Supplementary Fig. S2. Phenotypic correlations for all traits. LL (leaf length), LW (leaf width), FW (fruit weight), SW (stone weight) and FFPR (fruit flesh to pit ratio).

Supplementary Fig. S3. The amplified fragment size distributions of GBS libraries from olive genomic DNA digested with *Eco*T22I (a) and *Pst*I (b) restriction enzymes.

Supplementary Fig. S4. Histogram of missing marker data (a) and minor allele frequencies (b) in genotypes.

Supplementary Fig. S5. Delta K values over 10 runs.

Supplementary Fig. S6. Population structure of all accessions in the FULL panel.

Supplementary Fig. S7. Matrix showing pairwise Fst values between six groups.

Supplementary Fig. S8. Distribution of the r^2values of all (a) and $r^2 \geq 0.1$ (b) for all marker pairs in the FULL panel.

Supplementary Fig. S9. Comparison of QQ plots obtained using different number of PCs using MLM_K model for LL, LW, FW, SW and FFPR in the FULL panel.

Supplementary Fig. S10. QQ and Manhattan plots for LL, LW, FW, SW and FFPR in TOGR panel. The FDR significance threshold is shown in black.

Supplementary Fig. S11. QQ and Manhattan plots for LL, LW, FW, SW and FFPR in NCGR panel. The FDR significance threshold is shown in black.

Supplementary Tables S1-S13

Supplementary Table S1. Descriptive statistics and broad sense heritability (H^2) estimates of phenotypic data.

Supplementary Table S2. Pairwise-population genetic distance as calculated using Euclidean method. (see Excel file 'Supplementary Table S2.xlsx')

Supplementary Table S3. The distribution of the 183 olive accessions that shared at least 70% ancestry with one of the two inferred groups. (see Excel file 'Supplementary Table S13.xlsx')

Supplementary Table S4. Significant marker loci associated with LL, FW, SW and FFPR in the FULL panel and their chromosome location according to *Olea europaea* var. *Sylvestris* genome (Unver et al. 2017), P-value, major/minor alleles, proportion of phenotypic variation explained (R^2). U = genetic chromosomal location currently unknown.

Supplementary Table S5. Sequence read alignment (Blast+) of significant hits against *Olea europaea* var. *Sylvestris* genome (https://phytozome.jgi.doe.gov) (Unver et al. 2017).

Supplementary Table S6. Sequence read alignment (Blast+) of significant hits against *Olea europaea* L. subsp. *europaea* var. *europaea cv. 'Farga'* genome (Cruz et al. 2016).

Supplementary Table S7. Significant marker loci associated with LL, FW, SW and FFPR in TOGR accessions and their P-value, major/minor alleles, proportion of phenotypic variation explained (R^2).

Supplementary Table S8. Significant marker loci associated with LL, FW, SW and FFPR in NCGR accessions and their P-value, major/minor alleles, proportion of phenotypic variation explained (R^2).

Supplementary Table S9. Sequence read alignment (Blast+) of significant hits in TOGR accessions against *Olea europaea* var. *Sylvestris* genome (https://phytozome.jgi.doe.gov) (Unver et al. 2017).

Supplementary Table S10. Sequence read alignment (Blast+) of significant hits in TOGR accessions against *Olea europaea* L. subsp. *europaea* var. *europaea cv. 'Farga'* genome (Cruz et al. 2016).

Supplementary Table S11. Sequence read alignment (Blast+) of significant hits in NCGR accessions against *Olea europaea* var. *Sylvestris* genome (https://phytozome.jgi.doe.gov) (Unver et al. 2017).

Supplementary Table S12. Sequence read alignment (Blast+) of significant hits in NCGR accessions against *Olea europaea* L. subsp. *europaea* var. *europaea cv. 'Farga'* genome (Cruz et al. 2016).

Supplementary Table S13. List of olive genotypes used in the study. (see Excel file 'Supplementary Table S13.xlsx')

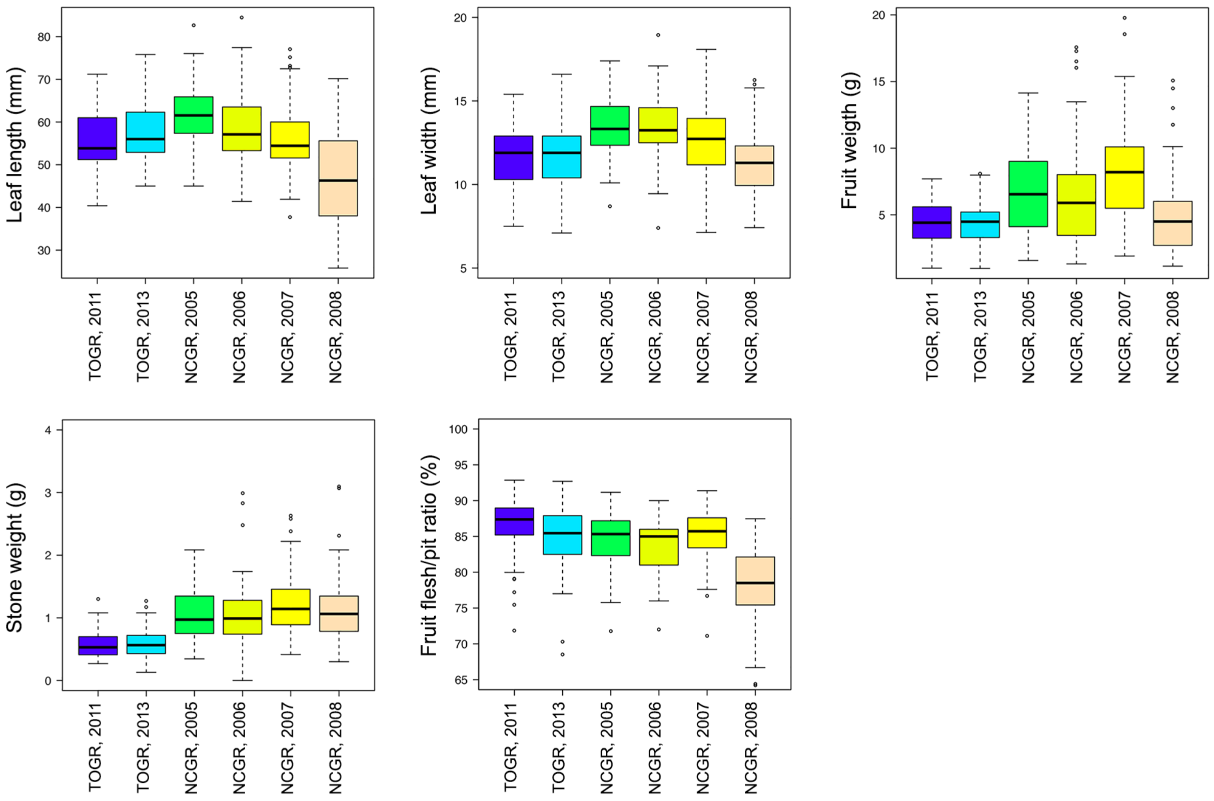

Supplementary Fig. S1. The box plots show the distribution of traits divided over geographical origin of accessions and year. TOGR (Turkish Olive GenBank Resource), NCGR (National Clonal Germplasm Repository).

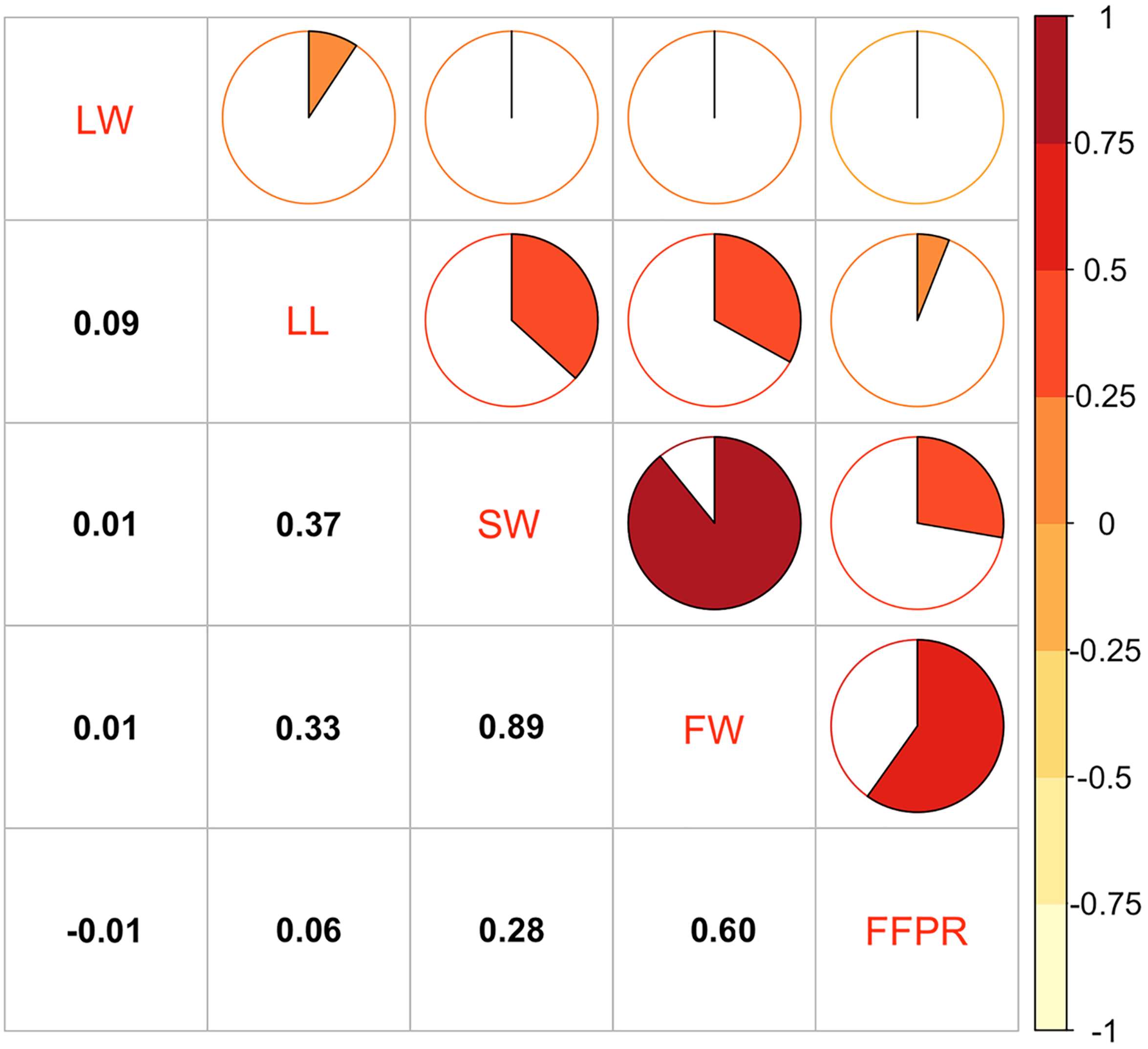

Supplementary Fig. S2. Phenotypic correlations for all traits. LL (leaf length), LW (leaf width), FW (fruit weight), SW (stone weight) and FFPR (fruit flesh to pit ratio).

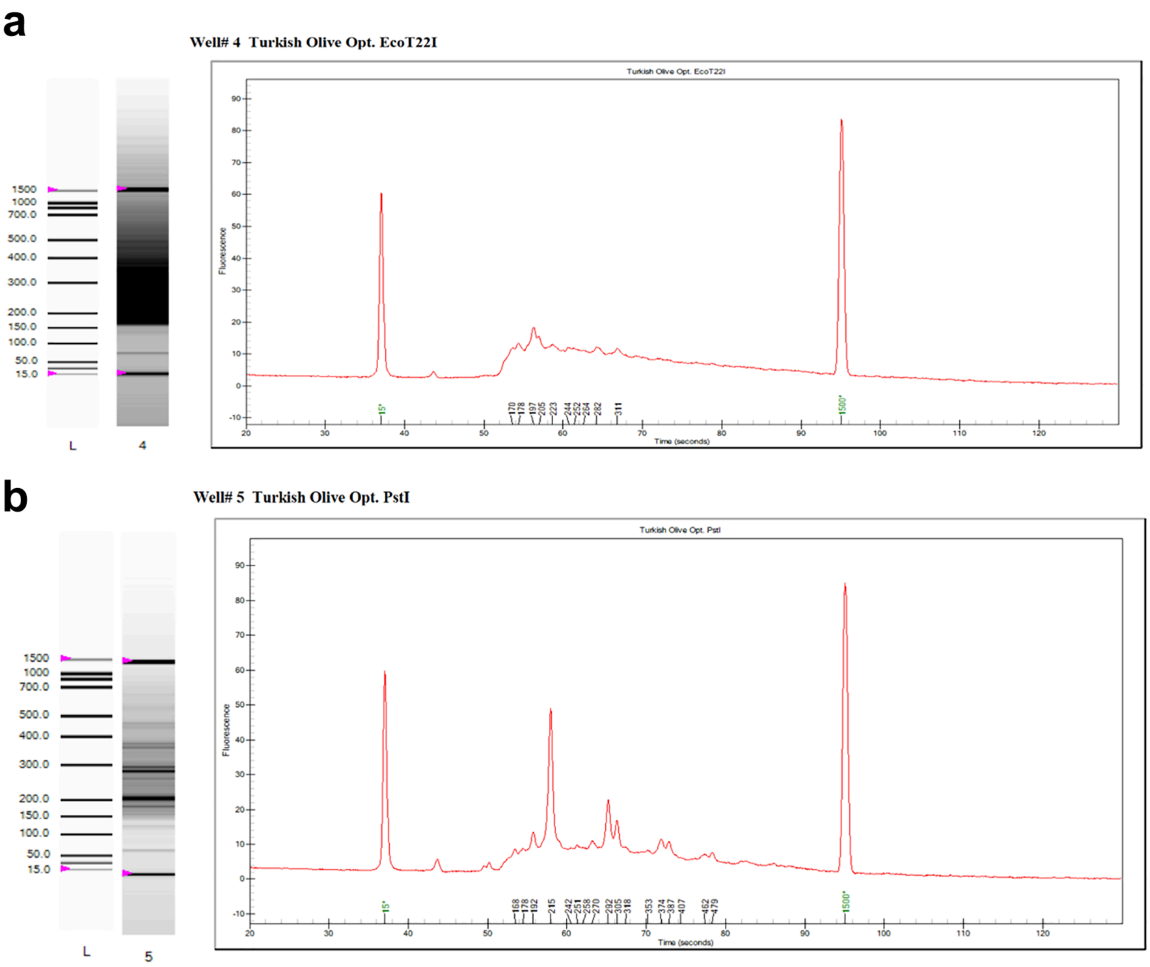

Supplementary Fig. S3. The amplified fragment size distributions of GBS libraries from olive genomic DNA digested with *Eco*T22I (a) and *Pst*I (b) restriction enzymes.

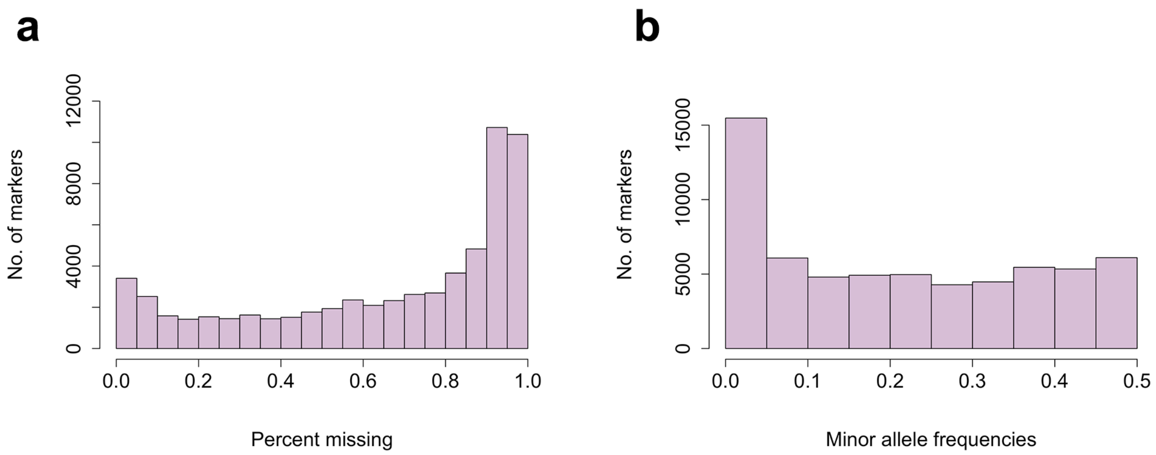

Supplementary Fig. S4. Histogram of missing marker data (a) and minor allele frequencies (b) in genotypes.

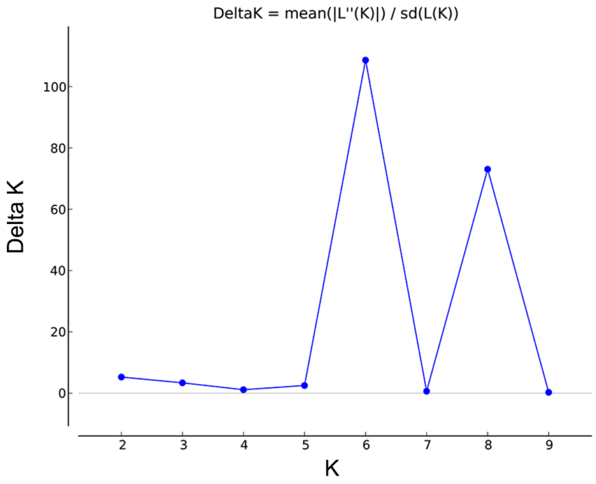

Supplementary Fig. S5. Delta K values over 10 runs.

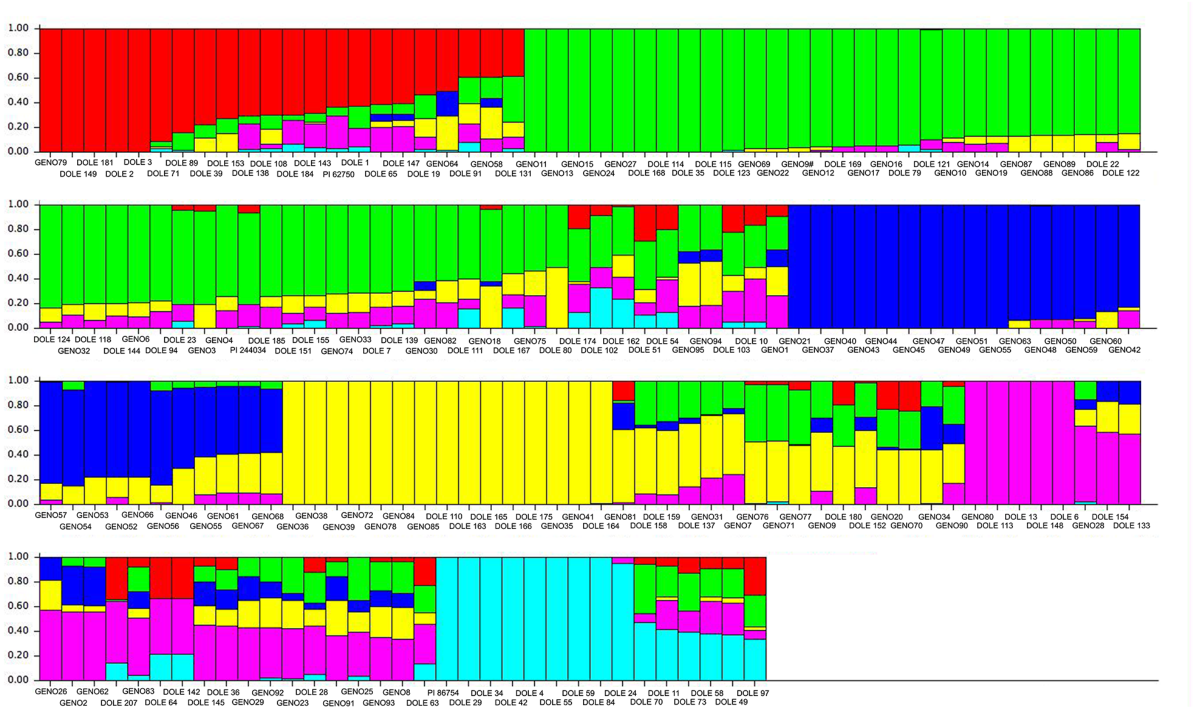

Supplementary Fig. S6. Population structure of all accessions in the FULL panel.

	Group 1	Group 2	Group 3	Group 4	Group 5	Group 6
Group 1						
Group 2	0.205					
Group 3	0.265	0.173				
Group 4	0.258	0.170	0.162			
Group 5	0.360	0.135	0.288	0.312		
Group 6	0.384	0.278	0.388	0.410	0.417	

Supplementary Fig. S7. Matrix showing pairwise Fst values between six groups.

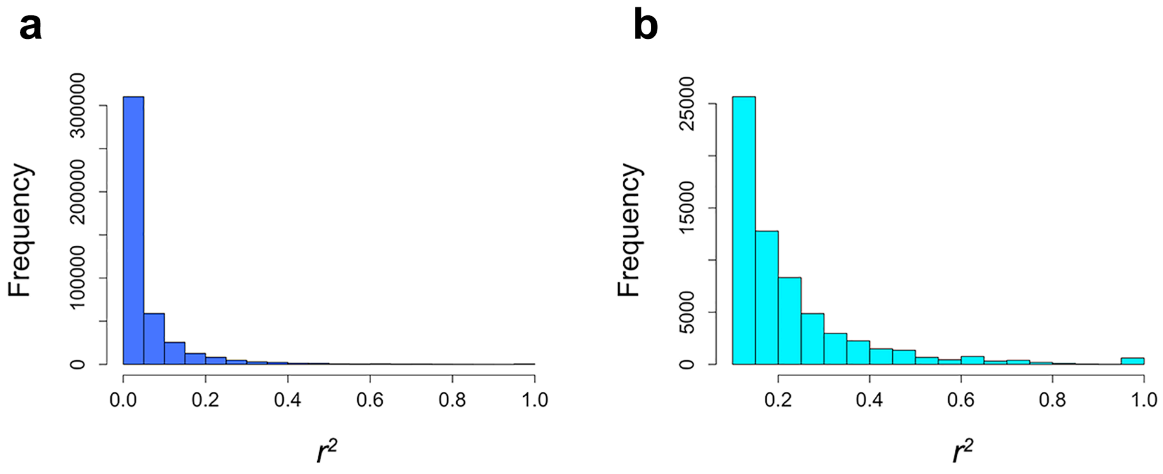

Supplementary Fig. S8. Distribution of the r^2values of all (a) and $r^2 \geq 0.1$ (b) for all marker pairs in the FULL panel.

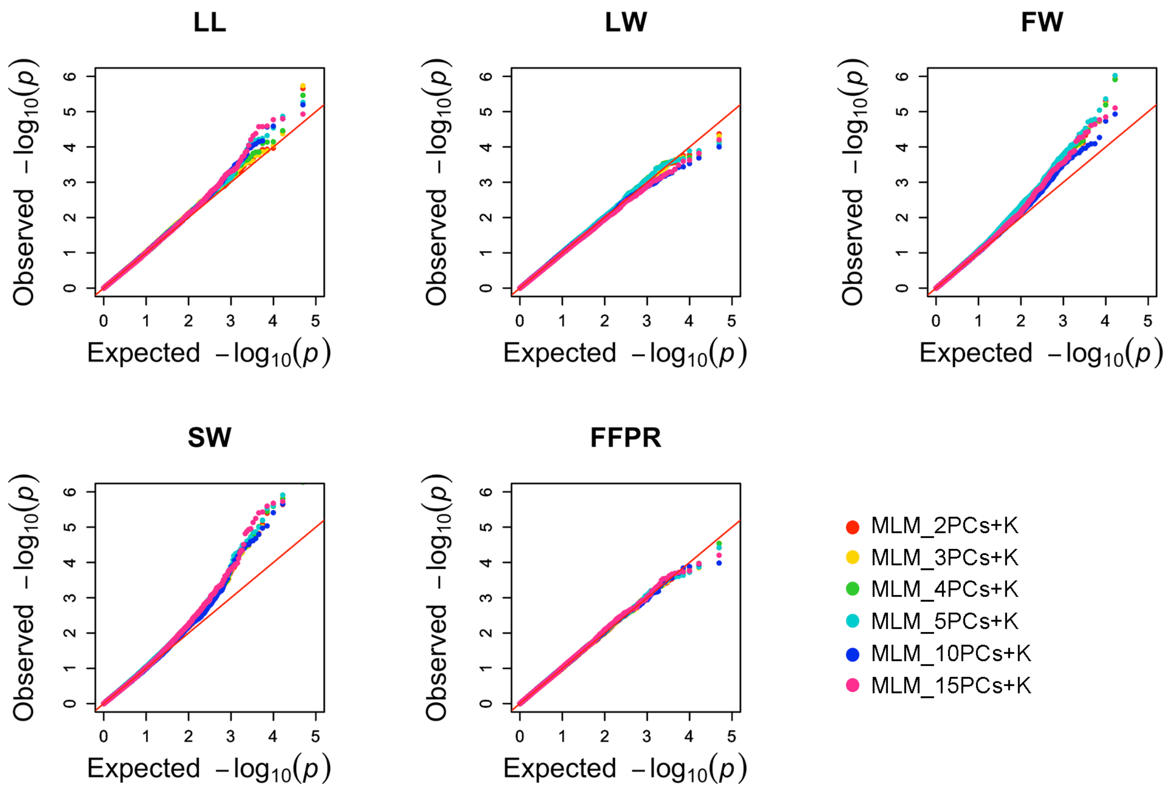

Supplementary Fig. S9. Comparison of QQ plots obtained using different number of PCs using MLM_K model for LL, LW, FW, SW and FFPR in the FULL panel.

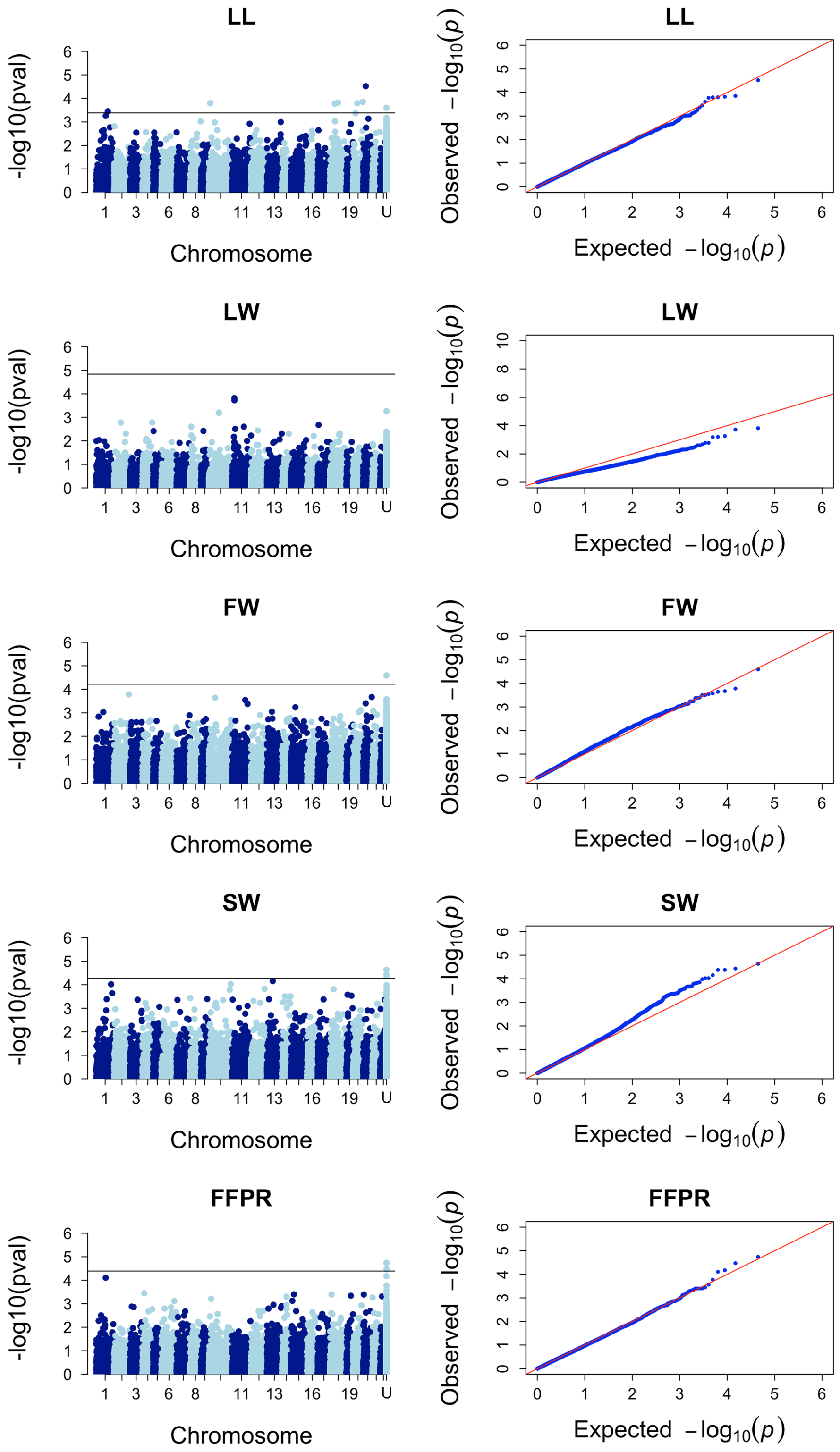

Supplementary Fig. S10. QQ and Manhattan plots for LL, LW, FW, SW and FFPR in TOGR panel. The FDR significance threshold is shown in black.

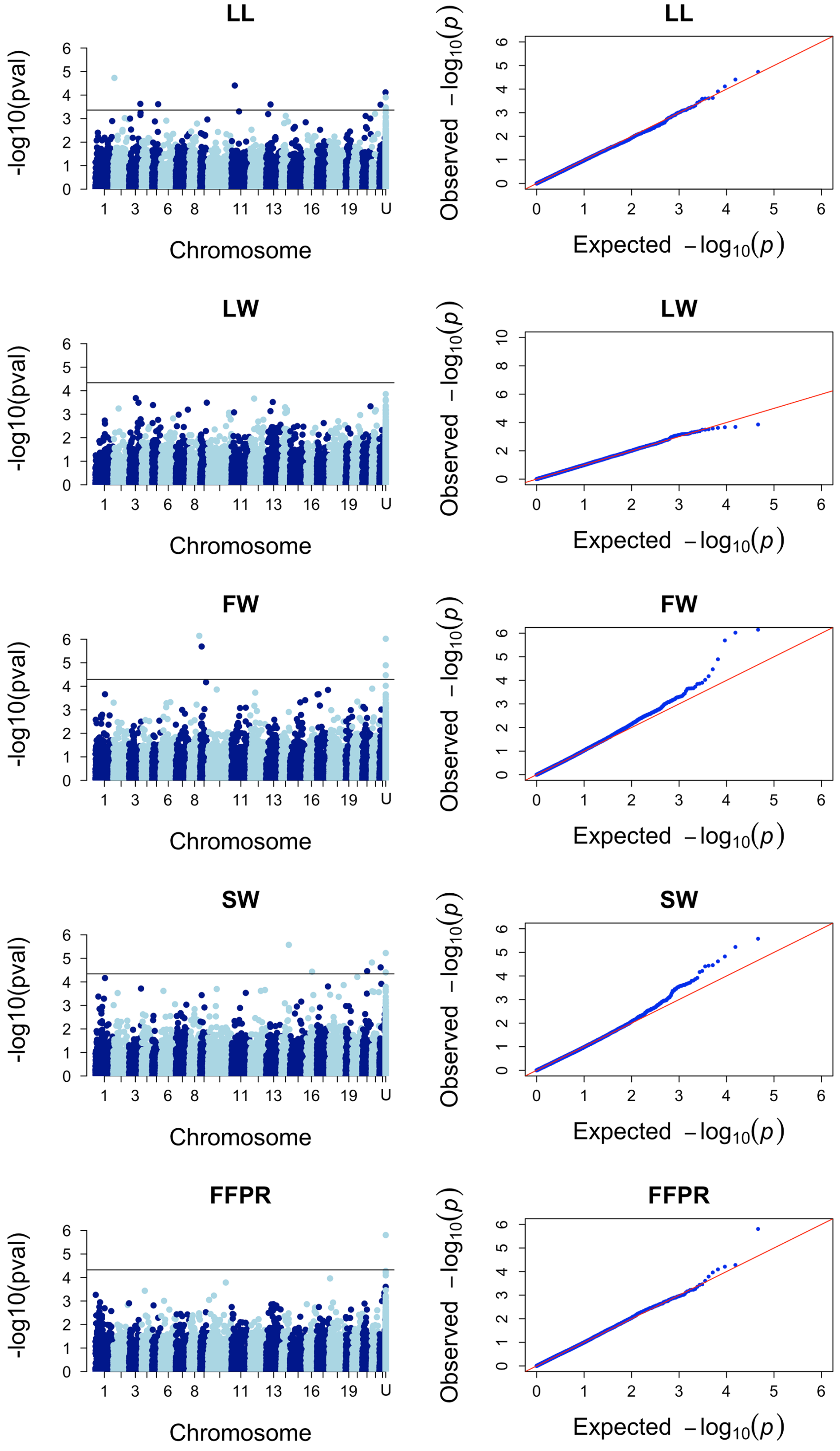

Supplementary Fig. S11. QQ and Manhattan plots for LL, LW, FW, SW and FFPR in NCGR panel. The FDR significance threshold is shown in black.

Supplementary Table S1. Descriptive statistics and broad sense heritability (H^2) estimates of phenotypic data.

	Min.	Max.	Median	Mean	Skew	Kurtosis	Variance	SD*	CV**	H^2***
LL	40.51	77.37	54.75	56.12	0.48	0.01	42.29	6.50	0.12	0.36
LW	7.30	26.10	12.19	12.20	1.58	10.72	4.13	2.03	0.17	0.52
FW	0.99	16.33	5.03	5.52	1.27	0.95	7.06	2.66	0.48	0.74
SW	0.21	4.72	0.75	0.86	3.05	17.32	0.26	0.51	0.60	0.73
FFPR	72.29	92.57	84.63	84.33	-0.69	0.26	13.49	3.67	0.04	0.43

* SD is the standard deviation of traits
** CV is the coefficient of variation of traits
*** Broad sense heritability of traits

Supplementary Table S4. Significant marker loci associated with LL, FW, SW and FFPR in the FULL panel and their chromosome location according to *Olea europaea* var. *Sylvestris* genome (Unver et al. 2017), P-value, major/minor alleles, proportion of phenotypic variation explained (R^2). U = genetic chromosomal location currently unknown.

Trait	Marker IDs	SNPs (Major/minor alleles)	Chromosome	P-value	FDR adjusted P-value	R^2
LL	S1_1842014	T/G	2	5.17E-04	5.17E-04	0.085
LL	S1_3166637	G/A	23	3.63E-05	7.41E-05	0.134
LL	S1_3833321	C/T	2	1.00E-06	1.33E-05	0.181
LL	S1_3864521	A/C	4	4.73E-04	4.82E-04	0.114
LL	S1_6490922	C/T	U	8.29E-05	1.13E-04	0.109
LL	S1_7655644	G/A	U	3.24E-04	3.44E-04	0.102
LL	S1_9001145	C/A	U	1.34E-04	1.51E-04	0.177
LL	S1_9282319	G/C	U	1.07E-04	1.32E-04	0.102
LL	S1_9768717	G/A	U	3.68E-04	3.83E-04	0.204
LL	S1_11667140	G/T	U	1.12E-04	1.32E-04	0.161
LL	S1_11738964	G/A	U	2.48E-04	2.68E-04	0.102
LL	S1_12793539	A/G	U	1.89E-04	2.08E-04	0.173
FW	S1_403246	T/A	9	6.56E-05	9.65E-05	0.053
FW	S1_407922	G/A	U	2.86E-05	7.23E-05	0.116
FW	S1_904125	T/A	10	1.63E-05	5.42E-05	0.070
FW	S1_1899635	T/C	U	8.96E-06	4.85E-05	0.196
FW	S1_4122458	T/A	11	2.83E-05	7.23E-05	0.123
FW	S1_4320026	A/G	U	1.30E-04	1.50E-04	0.154
FW	S1_4983712	T/C	U	1.04E-04	1.32E-04	0.137
FW	S1_5417823	G/A	U	8.28E-05	1.13E-04	0.114
FW	S1_6412238	T/G	6	9.88E-05	1.28E-04	0.163
FW	S1_6791646	T/A	U	5.04E-06	3.34E-05	0.224
FW	S1_6841033	C/A	U	3.63E-05	7.41E-05	0.158
FW	S1_7336035	C/T	15	5.53E-05	8.62E-05	0.232
FW	S1_7858740	C/T	1	5.23E-05	8.62E-05	0.274
FW	S1_9030959	T/C	8	4.97E-05	8.62E-05	0.113
FW	S1_10019163	C/A	19	5.76E-07	1.02E-05	0.153
FW	S1_11607122	C/T	15	1.10E-04	1.32E-04	0.109
FW	S1_12085523	G/A	11	4.10E-05	7.91E-05	0.070
FW	S1_12591134	G/T	U	4.08E-07	1.02E-05	0.278
FW	S1_13002224	C/T	11	3.02E-05	7.28E-05	0.129
SW	S1_904125	T/A	10	3.34E-05	7.41E-05	0.078
SW	S1_984251	G/A	18	1.99E-05	6.22E-05	0.200

SW	S1_1865851	A/C	6	1.23E-05	5.12E-05	0.104
SW	S1_1899635	T/C	U	6.01E-05	9.11E-05	0.193
SW	S1_2244037	T/C	18	4.18E-05	7.91E-05	0.105
SW	S1_3299322	G/T	U	2.25E-05	6.27E-05	0.087
SW	S1_4122458	T/A	11	8.52E-05	1.13E-04	0.132
SW	S1_6292562	C/A	22	1.26E-05	5.12E-05	0.080
SW	S1_7279745	A/T	U	1.55E-05	5.42E-05	0.131
SW	S1_7495520	A/T	23	5.43E-05	8.62E-05	0.081
SW	S1_8195416	A/C	23	1.55E-06	1.64E-05	0.102
SW	S1_9030959	T/C	8	1.14E-05	5.12E-05	0.156
SW	S1_11074838	G/T	12	9.15E-06	4.85E-05	0.063
SW	S1_12591134	G/T	U	4.73E-06	3.34E-05	0.274
SW	S1_13164923	C/T	21	7.04E-05	1.01E-04	0.179
SW	S1_13555831	C/T	12	2.68E-06	2.36E-05	0.067
SW	S1_13733736	G/A	U	2.20E-05	6.27E-05	0.143
SW	S1_13767032	G/A	16	9.11E-08	4.83E-06	0.198
FFPR	S1_138350	T/G	10	3.38E-05	7.41E-05	0.186
FFPR	S1_2068046	G/A	U	5.42E-05	8.62E-05	0.76
FFPR	S1_2372841	A/T	19	1.64E-05	5.42E-05	0.116
FFPR	S1_13164029	G/A	U	5.39E-05	8.62E-05	0.139

Supplementary Table S5. Sequence read alignment (Blast+) of significant hits against *Olea europaea* var. *Sylvestris* genome (https://phytozome.jgi.doe.gov) (Unver et al. 2017).

Marker IDs	Trait	Chromosome	Identity (%)	Alignment length	Sequence start	Sequence end	E-value
S1_1842014	LL	Chromosome 2	96.88	64	29785885	29785948	6.00E-23
S1_3833321	LL	Chromosome 2	100	64	2319724	2319787	3.00E-26
S1_3864521	LL	Chromosome 4	100	64	15689563	15689500	3.00E-26
S1_3166637	LL	Chromosome 23	100	64	2257995	2257932	3.00E-26
S1_7858740	FW	Chromosome 1	100	64	2178989	2178926	3.00E-26
S1_6412238	FW	Chromosome 6	98.44	64	13595315	13595252	1.00E-24
S1_403246	FW	Chromosome 9	96.83	63	3228516	3228454	2.00E-22
S1_12085523	FW	Chromosome 11	98.44	64	9794111	9794048	1.00E-24
S1_13002224	FW	Chromosome 11	100	64	15123146	15123083	3.00E-26
S1_11607122	FW	Chromosome 15	96.88	64	29979774	29979711	6.00E-23
S1_7336035	FW	Chromosome 15	96.36	55	20314526	20314472	6.00E-18
S1_10019163	FW	Chromosome 19	88.89	63	13098145	13098083	2.00E-13
S1_1865851	SW	Chromosome 6	100	64	5552372	5552435	3.00E-26
S1_11074838	SW	Chromosome 12	100	37	20703769	20703805	3.00E-11
S1_13555831	SW	Chromosome 12	92.98	57	28832680	28832736	1.00E-15
S1_13767032	SW	Chromosome 16	95.31	64	11635346	11635283	3.00E-21
S1_984251	SW	Chromosome 18	90.7	43	18570859	18570900	2.00E-07
S1_2244037	SW	Chromosome 18	98.44	64	1636301	1636238	1.00E-24
S1_13164923	SW	Chromosome 21	98.33	60	8831778	8831837	2.00E-22
S1_6292562	SW	Chromosome 22	100	61	677518	677578	1.00E-24
S1_7495520	SW	Chromosome 23	100	64	3840236	3840173	3.00E-26
S1_8195416	SW	Chromosome 23	100	64	2526292	2526355	3.00E-26
S1_138350	FFPR	Chromosome 10	98.44	64	34975953	34975890	1.00E-24
S1_2372841	FFPR	Chromosome 19	95.38	65	9186360	9186424	3.00E-21
S1_9030959	FW, SW	Chromosome 8	100	64	10401509	10401446	3.00E-26
S1_904125	FW, SW	Chromosome 10	98.44	64	37945231	37945168	1.00E-24
S1_4122458	FW, SW	Chromosome 11	98.44	64	15418699	15418636	1.00E-24

Supplementary Table S6. Sequence read alignment (Blast+) of significant hits against *Olea europaea* L. subsp. *europaea* var. *europaea cv. 'Farga'* genome (Cruz et al. 2016).

Marker IDs	Trait	Scaffold	Identity (%)	Alignment length	Sequence Start	Sequence end	E-value
S1_1842014	LL	ENA|FKYM01037411|FKYM01037411.1	100	64	34683	34746	6.00E-26
S1_3166637	LL	ENA|FKYM01021364|FKYM01021364.1	100	64	62740	62803	6.00E-26
S1_6490922	LL	ENA|FKYM01032078|FKYM01032078.1	100	64	37141	37204	6.00E-26
S1_7655644	LL	ENA|FKYM01033348|FKYM01033348.1	98.44	64	24514	24577	3.00E-24
S1_9001145	LL	ENA|FKYM01016414|FKYM01016414.1	98.44	64	3239	3176	3.00E-24
S1_11667140	LL	ENA|FKYM01042475|FKYM01042475.1	100	39	56817	56855	5.00E-12
S1_12793539	LL	ENA|FKYM01043772|FKYM01043772.1	100	64	18157	18220	6.00E-26
S1_403246	FW	ENA|FKYM01034877|FKYM01034877.1	98.44	64	26125	26062	3.00E-24
S1_4320026	FW	ENA|FKYM01050751|FKYM01050751.1	100	64	24407	24344	6.00E-26
S1_4983712	FW	ENA|FKYM01057319|FKYM01057319.1	98.44	64	7295	7232	3.00E-24
S1_5417823	FW	ENA|FKYM01053020|FKYM01053020.1	98.44	64	149259	149322	3.00E-24
S1_6412238	FW	ENA|FKYM01009744|FKYM01009744.1	96.88	64	13040	13103	1.00E-22
S1_6791646	FW	ENA|FKYM01019804|FKYM01019804.1	98.44	64	16755	16818	3.00E-24
S1_6841033	FW	ENA|FKYM01018037|FKYM01018037.1	94.74	38	48090	48053	4.00E-08
S1_7336035	FW	ENA|FKYM01024709|FKYM01024709.1	96.55	58	2131	2074	3.00E-19
S1_7858740	FW	ENA|FKYM01017775|FKYM01017775.1	100	64	7415	7352	6.00E-26
S1_10019163	FW	ENA|FKYM01011341|FKYM01011341.1	100	62	12317	12256	8.00E-25
S1_11607122	FW	ENA|FKYM01046637|FKYM01046637.1	100	64	22014	22077	6.00E-26
S1_12085523	FW	ENA|FKYM01037091|FKYM01037091.1	98.44	64	257	320	3.00E-24
S1_13002224	FW	ENA|FKYM01041072|FKYM01041072.1	100	64	13733	13670	6.00E-26
S1_984251	SW	ENA|FKYM01015267|FKYM01015267.1	89.36	47	3870	3824	4.00E-08
S1_1865851	SW	ENA|FKYM01053850|FKYM01053850.1	100	64	2496	2559	6.00E-26
S1_2244037	SW	ENA|FKYM01001624|FKYM01001624.1	98.44	64	53489	53426	3.00E-24
S1_3299322	SW	ENA|FKYM01041147|FKYM01041147.1	100	45	26472	26428	2.00E-15
S1_6292562	SW	ENA|FKYM01035965|FKYM01035965.1	100	61	575	515	3.00E-24

S1_7279745	SW	ENA|FKYM01011494|FKYM01011494.1	98.44	64	7205	7268	3.00E-24
S1_7495520	SW	ENA|FKYM01034874|FKYM01034874.1	100	64	17354	17291	6.00E-26
S1_8195416	SW	ENA|FKYM01034007|FKYM01034007.1	94.92	59	65996	65941	5.00E-17
S1_11074838	SW	ENA|FKYM01020464|FKYM01020464.1	100	37	159	195	6.00E-11
S1_13164923	SW	ENA|FKYM01027617|FKYM01027617.1	97.87	47	10466	10420	8.00E-15
S1_13555831	SW	ENA|FKYM01050895|FKYM01050895.1	96.61	59	29397	29339	8.00E-20
S1_13733736	SW	ENA|FKYM01045820|FKYM01045820.1	98.44	64	3279	3216	3.00E-24
S1_13767032	SW	ENA|FKYM01020795|FKYM01020795.1	96.88	64	44372	44435	1.00E-22
S1_138350	FFPR	ENA|FKYM01000971|FKYM01000971.1	98.44	64	45461	45524	3.00E-24
S1_2372841	FFPR	ENA|FKYM01053865|FKYM01053865.1	100	64	1073	1136	6.00E-26
S1_13164029	FFPR	ENA|FKYM01015092|FKYM01015092.1	98.44	64	49346	49283	3.00E-24
S1_1899635	FW, SW	ENA|FKYM01056855|FKYM01056855.1	98.44	64	21878	21941	3.00E-24
S1_4122458	FW, SW	ENA|FKYM01026187|FKYM01026187.1	98.44	64	19254	19317	3.00E-24
S1_9030959	FW, SW	ENA|FKYM01025953|FKYM01025953.1	100	64	17346	17283	6.00E-26
S1_12591134	FW, SW	ENA|FKYM01014866|FKYM01014866.1	100	33	81948	81980	1.00E-08

Supplementary Table S7. Significant marker loci associated with LL, FW, SW and FFPR in TOGR accessions and their P-value, major/minor alleles, proportion of phenotypic variation explained (R^2).

Trait	Marker	SNPs (major/minor alleles)	P-value	FDR adjusted P-value	R^2 (%)
LL	S1_277218	G/A	3.03E-05	6.92E-05	0.23681
LL	S1_3758459	A/G	0.000160107	0.000211382	0.13817
LL	S1_4516750	G/A	0.000250577	0.000278588	0.18490
LL	S1_8995538	G/T	0.000140586	0.000205472	0.19755
LL	S1_11033062	G/A	0.000151913	0.000211382	0.19395
LL	S1_11985416	T/C	0.000161318	0.000211382	0.30158
LL	S1_12881158	A/C	0.000353186	0.000362731	0.24399
LL	S1_13394433	C/T	0.000169403	0.000214578	0.28068
FW	S1_3288416	G/A	2.56E-05	6.92E-05	0.11796
SW	S1_7180344	A/G	2.31E-05	6.92E-05	0.31786
SW	S1_7906257	G/C	4.19E-05	6.92E-05	0.39933
SW	S1_11265724	G/T	4.18E-05	6.92E-05	0.38549
SW	S1_11920945	G/T	3.66E-05	6.92E-05	0.34438
FFPR	S1_4640124	G/C	1.82E-05	6.92E-05	0.25531
FFPR	S1_7559040	G/T	3.40E-05	6.92E-05	0.20400

Supplementary Table S8. Significant marker loci associated with LL, FW, SW and FFPR in NCGR accessions and their P-value, major/minor alleles, proportion of phenotypic variation explained (R^2).

Trait	Marker	SNPs (major/minor alleles)	P-value	FDR adjusted P-value	R^2 (%)
LL	S1_3166637	G/A	0.000256594	0.000278588	0.18850
LL	S1_3296027	T/C	0.000244692	0.000278588	0.32743
LL	S1_3833321	C/T	1.86E-05	6.92E-05	0.23589
LL	S1_6490922	C/T	0.000125728	0.000191107	0.16229
LL	S1_7192958	T/C	0.000250502	0.000278588	0.27654
LL	S1_11298821	G/A	7.69E-05	0.000121689	0.25007
LL	S1_11738964	G/A	0.000377516	0.000377516	0.17102
LL	S1_11740924	G/T	0.000334111	0.000352673	0.16463
LL	S1_13291221	A/T	3.93E-05	6.92E-05	0.36425
LL	S1_13355714	T/C	0.000236267	0.000278588	0.16116
FW	S1_403246	T/A	2.03E-06	1.93E-05	0.13657
FW	S1_991244	C/A	3.40E-05	6.92E-05	0.20078
FW	S1_6841033	C/A	9.54E-07	1.81E-05	0.29980
FW	S1_12591134	G/T	1.28E-05	6.92E-05	0.31092
FW	S1_13473561	C/T	7.15E-07	1.81E-05	0.17978
SW	S1_6292562	C/A	1.50E-05	6.92E-05	0.13950
SW	S1_6841033	C/A	5.93E-06	3.75E-05	0.32527
SW	S1_8195416	A/C	2.42E-05	6.92E-05	0.12592
SW	S1_8558531	T/A	3.94E-05	6.92E-05	0.13199
SW	S1_9345239	C/T	2.65E-06	2.02E-05	0.18649
SW	S1_12485231	G/A	3.51E-05	6.92E-05	0.12669
SW	S1_13767032	G/A	3.67E-05	6.92E-05	0.17913
FFPR	S1_10335130	T/C	1.57E-06	1.93E-05	0.38026

Supplementary Table S9. Sequence read alignment (Blast+) of significant hits in TOGR accessions against *Olea europaea* var. *Sylvestris* genome (https://phytozome.jgi.doe.gov) (Unver et al. 2017).

Marker IDs	Trait	Chromosome	Identity (%)	Alignment length	Sequence start	Sequence end	E-value
S1_12881158	LL	Chromosome 1	100	64	22664876	22664939	3.00E-26
S1_3758459	LL	Chromosome 10	95.31	64	2748598	2748661	3.00E-21
S1_11033062	LL	Chromosome 18	100	61	15752581	15752521	1.00E-24
S1_13394433	LL	Chromosome 18	98.44	64	9322147	9322210	1.00E-24
S1_8995538	LL	Chromosome 20	100	64	19319680	19319617	3.00E-26
S1_11985416	LL	Chromosome 20	98.31	59	8458906	8458964	8.00E-22
S1_277218	LL	Chromosome 21	98.44	64	5110201	5110138	1.00E-24

Supplementary Table S10. Sequence read alignment (Blast+) of significant hits in TOGR accessions against *Olea europaea* L. subsp. *europaea* var. *europaea cv. 'Farga'* genome (Cruz et al. 2016).

Marker IDs	Trait	Scaffold	Identity (%)	Alignment length	Sequence start	Sequence end	E-value
S1_277218	LL	ENA|FKYM01014887|FKYM01014887.1	91.53	59	18136	18194	8.00E-15
S1_3758459	LL	ENA|FKYM01036060|FKYM01036060.1	100	64	59447	59510	6.00E-26
S1_4516750	LL	ENA|FKYM01010929|FKYM01010929.1	98.44	64	7868	7931	3.00E-24
S1_8995538	LL	ENA|FKYM01012157|FKYM01012157.1	100	64	17529	17592	6.00E-26
S1_11985416	LL	ENA|FKYM01030510|FKYM01030510.1	100	64	1621	1684	6.00E-26
S1_12881158	LL	ENA|FKYM01039655|FKYM01039655.1	100	64	77309	77372	6.00E-26
S1_13394433	LL	ENA|FKYM01039636|FKYM01039636.1	100	64	13807	13870	6.00E-26
S1_3288416	FW	ENA|FKYM01000920|FKYM01000920.1	100	64	47974	48037	6.00E-26
S1_7180344	SW	ENA|FKYM01004983|FKYM01004983.1	98.44	64	83286	83224	1.00E-23
S1_7906257	SW	ENA|FKYM01004773|FKYM01004773.1	100	56	26633	26688	2.00E-21
S1_11265724	SW	ENA|FKYM01013085|FKYM01013085.1	98.44	64	10998	11061	3.00E-24
S1_11920945	SW	ENA|FKYM01031867|FKYM01031867.1	96.88	64	10711	10774	1.00E-22
S1_4640124	FFPR	ENA|FKYM01003062|FKYM01003062.1	100	64	34126	34189	6.00E-26
S1_7559040	FFPR	ENA|FKYM01031349|FKYM01031349.1	100	39	19163	19125	5.00E-12

Supplementary Table S11. Sequence read alignment (Blast+) of significant hits in NCGR accessions against *Olea europaea* var. *Sylvestris* genome (https://phytozome.jgi.doe.gov) (Unver et al. 2017).

Marker IDs	Trait	Chromosome	Identity (%)	Alignment length	Sequence start	Sequence end	E-value
S1_13355714	LL	Chromosome 3	98.44	64	22353356	22353419	1.00E-24
S1_3296027	LL	Chromosome 5	96.88	64	11788786	11788849	6.00E-23
S1_13291221	LL	Chromosome 11	87.5	64	6733796	6733859	6.00E-13
S1_7192958	LL	Chromosome 13	93.1	58	8757142	8757085	3.00E-16
S1_11298821	LL	Chromosome 23	98.18	55	12096948	12096894	1.00E-19
S1_13473561	FW	Chromosome 8	98.39	62	19710831	19710770	2.00E-23
S1_403246	FW	Chromosome 9	96.83	63	3228516	3228454	2.00E-22
S1_9345239	SW	Chromosome 14	96.88	64	14932685	14932746	2.00E-22
S1_13767032	SW	Chromosome 16	95.31	64	11635346	11635283	3.00E-21
S1_12485231	SW	Chromosome 21	98.44	64	9406435	9406372	1.00E-24
S1_6292562	SW	Chromosome 22	100	61	677518	677578	1.00E-24
S1_8195416	SW	Chromosome 23	100	64	2526292	2526355	3.00E-26

Supplementary Table S12. Sequence read alignment (Blast+) of significant hits in NCGR accessions against *Olea europaea* L. subsp. *europaea* var. *europaea cv. 'Farga'* genome (Cruz et al. 2016).

Marker IDs	Trait	Scaffold	Identity (%)	Alignment length	Sequence start	Sequence end	E-value
S1_3296027	LL	ENA|FKYM01052148|FKYM01052148.1	98.39	62	8135	8074	4.00E-23
S1_3833321	LL	ENA|FKYM01012220|FKYM01012220.1	98.44	64	39760	39823	3.00E-24
S1_7192958	LL	ENA|FKYM01051077|FKYM01051077.1	96.72	61	1572	1512	6.00E-21
S1_11298821	LL	ENA|FKYM01004833|FKYM01004833.1	98.44	64	33572	33635	3.00E-24
S1_13291221	LL	ENA|FKYM01020878|FKYM01020878.1	100	64	47353	47290	6.00E-26
S1_13355714	LL	ENA|FKYM01000932|FKYM01000932.1	98.44	64	95214	95151	3.00E-24
S1_13473561	FW	ENA|FKYM01043802|FKYM01043802.1	100	64	54595	54532	6.00E-26
S1_8558531	SW	ENA|FKYM01053801|FKYM01053801.1	98.44	64	114378	114315	3.00E-24
S1_9345239	SW	ENA|FKYM01041474|FKYM01041474.1	96.88	64	88151	88212	5.00E-22
S1_12485231	SW	ENA|FKYM01027285|FKYM01027285.1	98.44	64	994	1057	3.00E-24
S1_10335130	FFPR	ENA|FKYM01009909|FKYM01009909.1	96.88	64	18434	18371	1.00E-22
